# Modulating mechanical stability of heterodimerization between engineered orthogonal helical domains

**DOI:** 10.1038/s41467-020-18323-w

**Published:** 2020-09-08

**Authors:** Miao Yu, Zhihai Zhao, Zibo Chen, Shimin Le, Jie Yan

**Affiliations:** 1 Mechanobiology Institute, National University of Singapore, Singapore, 117411 Singapore; 2 Department of Physics, National University of Singapore, Singapore, 117542 Singapore; 3 Department of Biochemistry, University of Washington, Seattle, WA 98195 USA

**Keywords:** Magnetic tweezers, Single-molecule biophysics, Protein design, Protein folding

## Abstract

Mechanically stable specific heterodimerization between small protein domains have a wide scope of applications, from using as a molecular anchorage in single-molecule force spectroscopy studies of protein mechanics, to serving as force-bearing protein linker for modulation of mechanotransduction of cells, and potentially acting as a molecular crosslinker for functional materials. Here, we explore the possibility to develop heterodimerization system with a range of mechanical stability from a set of recently engineered helix-heterotetramers whose mechanical properties have yet to be characterized. We demonstrate this possibility using two randomly chosen helix-heterotetramers, showing that their mechanical properties can be modulated by changing the stretching geometry and the number of interacting helices. These helix-heterotetramers and their derivatives are sufficiently stable over physiological temperature range. Using it as mechanically stable anchorage, we demonstrate the applications in single-molecule manipulation studies of the temperature dependent unfolding and refolding of a titin immunoglobulin domain and α-actinin spectrin repeats.

## Abstract (web summary)

Mechanically stable specific heterodimerization formed with reversible bonds are used as a molecular anchorage in single-molecule force spectroscopy studies with unique mechanical properties. Here authors develop a variety of heterodimerization molecular systems with a range of mechanical stability from a set of recently engineered helix-heterotetramers.

## Introduction

Specific heterodimerization systems formed with rapid reversible bonds that can withstand physiological level of mechanical forces (within tens of piconewtons) over physiological time scales (typically within minutes)^1–7^ have a wide scope of applications in biological, biophysical, biomedical, and biomaterial studies^8–22^. For example, substitution of native force-bearing ligand–receptor interactions by mechanically stable heterodimerization systems has revealed how the notch signaling pathway and T-cells can be mechanically activated^17,18,20^. They have also been extensively used as crosslinkers of biopolymers to develop biocompatible hydrogels with unique mechanical properties that have important biomedical applications^15,19,21,22^. In single-molecule mechanical studies of force-dependent structural changes and interactions of biomolecules, these systems have also been utilized as specific mechanically stable molecular anchorage to form single-molecule tethers under force^9–14,16,23–28^.

Currently available mechanically stable and reversible heterodimerization systems can be broadly categorized into several groups: (1) Streptavidin–biotin interaction-based systems^8–14,16,24–31^, (2) Antibody–antigen interaction-based systems^12,13,23,25,32,33^, (3) DNA hybridization-based systems^15,17,21^, and (4) Coiled-coil motif-based systems^34,35^. Streptavidin–biotin systems have been widely used in single-molecule manipulation experiments^8–14,16,23–28,31^ due to the rapid formation of the bond and its high mechanical stability at typical room temperatures^36^. Two representative antibody–antigen systems based on digoxigenin–antidigoxygenin or GST–antiGST interactions have been frequently used as a mechanical anchorage^12,13,25,32,33^. Despite high affinity and high specificity, these antigen–antibody systems have lower mechanical stability than streptavidin–biotin interaction^37,38^. The DNA hybridization-based system has a unique advantage in that its mechanical stability can be programmed over a wide range by modulating the length, sequence and the force geometry applied to the hybrid^17,39,40^. However, as the DNA is a highly negatively charged molecule, potential non-specific interactions may occur between DNA hybrid and other counter-charged factors. The coiled-coil motif-based system have the advantage of having small molecular weight and can be easily fused to target proteins^34,35,41–43^. The currently coiled-coil-based systems typically rupture at forces of 10–40 pN at high loading rate (~1000 pN s^−1^) in atomic force microscopy experiment^44,45^, which is comparable to the mechanical stability of digoxygenin–antidigoxygenin complex^37^.

The mechanical stability of heterodimerization systems can vary over a broad temperature range, depending on the nature of the dominant interactions that hold the complex together. In general, the strength of exothermic bonds such as hydrogen bonds decreases as temperature increases^46^, while the strength of endothermic bonds such as many of the entropy-driven hydrophobic interactions increases as temperature increases^46^. The mechanical lifetime of most currently applied heterodimerization systems, such as streptavidin–biotin bond and DNA hybrid, rapidly decreases as temperature increases^29,39,47^, which imposes a strong restriction of their applications in studies involving a significant range of temperature changes.

**Fig. 1:**
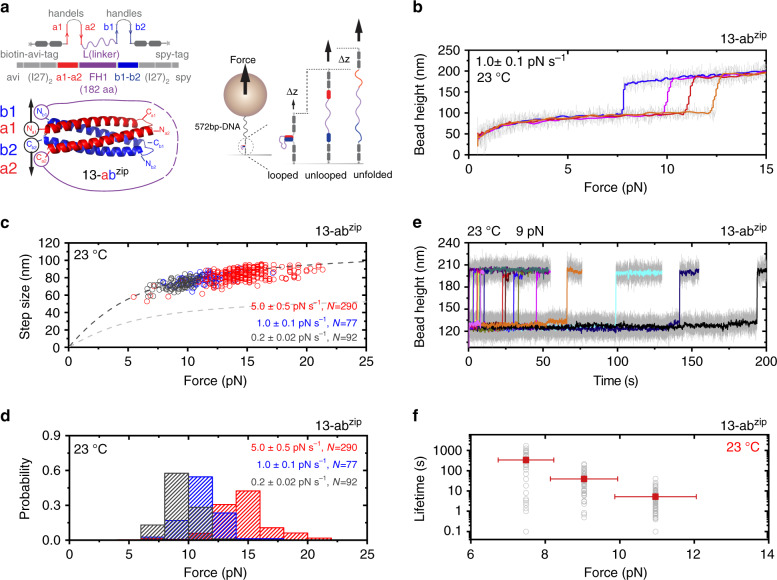
Single-molecule quantification of the mechanical stability of 13-ab^zip^. **a** Left-top panel: design of the 13-a1a2Lb1b2-construct. Left-bottom panel: the expected structure of 13-ab^zip^. Black circles and black arrows indicate the force-attaching points and force direction. Purple circles indicate the attachment points of the long linker. Components a and b are helix-hairpins that contains two helices a1&a2 (red), and b1&b2 (blue), respectively. Right panel: sketch of a single-molecule construct specifically tethered between a coverslip surface and a superparamagnetic bead with a 572-bp DNA handle (Supplementary Note 2). **b** Four representative force–height curves of the single-molecule 13-a1a2Lb1b2-construct at 1.0 ± 0.1 pN s^−1^. The stepwise bead height jump indicates the force-induced rupturing (unlooping) of the 13-ab^zip^. **c** The force–step size graph of the force-dependent rupturing (unlooping) transitions of the 13-ab^zip^. The dash curves are the theoretically predicted. The dark gray line is predicted assuming that in the unlooped state a and b components are unfolded into an unstructured peptide chain; the light gray line is predicted assuming that the a and b components remain as folded helix-hairpin structures (Supplementary Note 6 and Supplementary Figs. 4 and 5). **d** Normalized histograms of the 13-ab^zip^ rupture forces. **e** Representative time traces of the bead height before and after the rupture of the 13-ab^zip^ at ~9 pN, where the rupturing events are indicated by the large bead height jump steps. **f** Force-dependent lifetimes of the 13-ab^zip^. The solid squares represent the average lifetimes (*τ*) obtained by best-fitting of the lifetime histogram to a single-exponential decay function. The hollow gray circles represent individual lifetimes (*N* ~ 100 for each force). The vertical error bars indicate the standard errors of the lifetime obtained by $\frac{\tau }{{\sqrt {N - 1} }}$. The horizontal error bars indicate 10% of relative force uncertainty resulted from the force-calibration method. For panels **b**–**f**: the experiments were performed at 23 °C. For panels **b** and **e**: 20-points FFT smooth (colored lines) of the raw data (gray lines) are shown. For panels **c**, **d**: the number of data points obtained from >5 independent tethers are indicated. Source data are provided as a Source Data file.

Therefore, there is a need to develop heterodimerization systems that retain sufficient mechanical stability at physiological temperature range using small protein domains that can be easily fused with the target. Recent studies reported orthogonal heterodimerization between two helix-forming motifs labeled as a and b (Fig. 1a), each being designed to form a helix-hairpin. The interactions between these helix-forming motifs, which result in formation of four-helix bundles (hereafter referred to as helix-heterotetramers) with high thermal stability, are optimized for high-affinity orthogonal inter-helix interactions by computational models^43,48,49^ (Fig. 1a). The orthogonality of these heterodimerization systems makes it appealing to develop a variety of mechanically stable heterodimerization systems from them. However, whether these helix-heterotetramers have enough high mechanical stability over physiological temperature range is unclear.

In this study, we quantified the temperature-dependent mechanical stability of two randomly chosen heterodimerization systems (#13 and #37) from the reported list^49^ and found that the resulting helix-heterotetramers are sufficiently mechanically stable over physiological temperature range. We further show that mechanical stability of the formed helix-heterotetramers can be changed by modulating the stretching force geometry and the number of interacting helices. We demonstrate the applications of the helix-heterotetramer system as mechanically stable anchorage for single-molecule mechanical manipulation studies of the protein domain unfolding/refolding dynamics and domain stability over physiological temperature range.

## Results

### The mechanical stability of the helix-heterotetramer

We developed a single-molecule assay using our magnetic-tweezer setup^50–53^ to directly quantify the loading rate-dependent rupture forces and the force-dependent lifetime of the helix-heterotetramers (Supplementary Notes 1–2). Briefly, the single-molecule construct essentially contains the complementary components of the helix-heterotetramer (hereafter referred to as a and b), linked by a long flexible unstructured peptide chain (L) (Fig. 1a left panel, and Supplementary Fig. 1a). The components a and b, each containing two helices a1, a2 and b1, b2, respectively, are expected to form two helix-hairpins by design^49^. The resulting complex by a and b is, therefore, a helix-heterotetramer. When a and b are separated under force, the linker L keeps them in close vicinity to allow reformation of the complex by dropping the force. In addition, the long linker gives rise to a large extension difference between the separated and associated states of the two components that can be unambiguous distinguished from domain unfolding. Hence, the linker increases both the experimental throughput and the detection accuracy. Importantly, as the peptide linker has a very low bending persistence length, it does not introduce significant mechanical perturbation to the helix-heterotetramers (Supplementary Note 3). The linked a and b (a-L-b) in the construct is spanned between two repeats of the well-characterized titin I27 domain^27^ at each side, which serve as molecular spacers and specificity control. The N- and C- termini of the construct contain a biotin-avi-tag and a spy-tag, respectively, which enables specific tethering for the single-molecule force spectroscopy experiments (Supplementary Notes 1–2).

The linked complementary a and b helix-hairpins can form a helix-heterotetramer at low forces, looping the linker (referred to as the looped state). Rupture of the helix-heterotetramer under force leads to release of the linker, accompanied with a stepwise extension increase (referred to as the unlooped state). As the linker has >200 residues, this stepwise extension can be easily differentiated from unfolding of the 89 residues of I27 domain in the handle (Supplementary Note 4)^31^. The looped and unlooped states can be tuned by force (Fig. 1a, right panel). Since the a and b components in the construct are linked in such a way where the force is applied to the N-terminus of the a1 helix in the a component and the C-terminus of the b2 helix in the b component, we refer the protein construct to as a1a2Lb1b2 (left-to-right: N-to-C-termini). The way of force attaching to the construct results in an unzipping force geometry when the helix-heterotetramer forms (Fig. 1a left panel, and Supplementary Fig. 1a); therefore, we refer to the helix-heterotetramer in this construct as ab^zip^.

In typical single-molecule magnetic-tweezer experiments, a molecule is either subjected to a time-varying force or a constant force, referred to as force-loading or force-clamping experiments, respectively. In the former, the force at which the structural transition of the molecule occurs is recorded, which depends on the force-loading rate. In the latter, the time taken to the transition is recorded, which depends on the level of the applied force. We investigated the force-response of the 13-ab^zip^ under both force-loading and constant force constraints, where the prefix 13 indicates the #13 helix-heterotetramer in the reported list^49^.

For the force-loading experiments, we held the construct at sufficiently low force to allow the formation of the looped state and then linearly increased the force with a loading rate, and recorded the rupture force at which unlooping occurred. By repeating the force-loading procedure for sufficient number of cycles, we obtained the distribution of the rupture forces of the 13-ab^zip^ helix-heterotetramer at given force-loading rates. We observed characteristic stepwise unfolding signals from the a and b components for the unlooped 13-ab^zip^ construct at forces within 9 pN at a loading rate of 1 pN s^−1^, associated with step sizes ~12 nm (Supplementary Fig. 2 and Supplementary Note 5). This result suggests that the helix-forming motifs in a and b indeed form stable helix-hairpins, which further interact with each other to form the helix-heterotetramer. For the quantification of the force-dependent lifetime, after formation of the 13-ab^zip^ helix-heterotetramer at low forces, we directly jumped to different levels of forces and measured the time duration until unlooping occurred. By repeating the force-jumping procedure for a sufficient number of cycles at multiple force levels, we obtained the statistics of the force-dependent lifetime of the 13-ab^zip^ helix-heterotetramer.

Figure 1b shows the representative force-bead height curves of the a1a2Lb1b2-construct from ~1 to ~20 pN measured with a loading rate of 1 pN s^−1^. Each colored curve represents one independent force-increase loading cycle. The abrupt stepwise bead height jump in each curve indicates the force-dependent rupturing of the 13-ab^zip^ helix-heterotetramer and the resulting unlooping. The rupturing of the 13-ab^zip^ helix-heterotetramer and the unfolding of the separated a and b helix-hairpins typically occurred concurrently because the unfolding forces of the a and b helix- hairpins (<9 pN) are smaller than the unlooping forces (Supplementary Fig. 2 and Supplementary Note 5). Here, we note that in such force-loading experiments, due to the small transition distance of I27 (~0.6 nm^27^), I27 retains its low unfolding rate (~10^−3^ s^−1^) over the scanned force range up to 50 pN^31,54^. Hence, I27 unfolding was not observed in such force-loading experiments (Supplementary Figs. 3–5 and Supplementary Note 4).

Figure 1c shows force–step size graph of the force-dependent rupture transitions of the 13-ab^zip^ helix-heterotetramer at three loading rates of 0.2 pN s^−1^ (dark gray), 1 pN s^−1^ (blue) and 5 pN s^−1^ (red). The corresponding normalized distributions of the rupture forces shows Gaussian-like distributions with peak values of ~8, ~12, and ~15 pN, respectively (Fig. 1d). Figure 1e shows examples of the bead height time traces of the a1a2Lb1b2-construct at 9 pN during force-clamping experiments (more representative time traces are provided in Supplementary Fig. 6). The ~80 nm height increase steps are from the force-induced rupturing of 13-ab^zip^ helix-heterotetramer that causes unlooping and concurrent unfolding of the a and b helix-hairpins obtained from different force-clamping cycles. Figure 1f shows the force-dependent average lifetimes of the 13-ab^zip^ helix-heterotetramer at different forces obtained by fitting the histogram of the measured lifetime data to a single-exponential decay function (Supplementary Fig. 7). The data show that the 13-ab^zip^ helix-heterotetramer can withstand forces of 5–12 pN for seconds to minutes depending on the force level.

### Modulating the mechanical stability of the helix-bundles

**Fig. 2:**
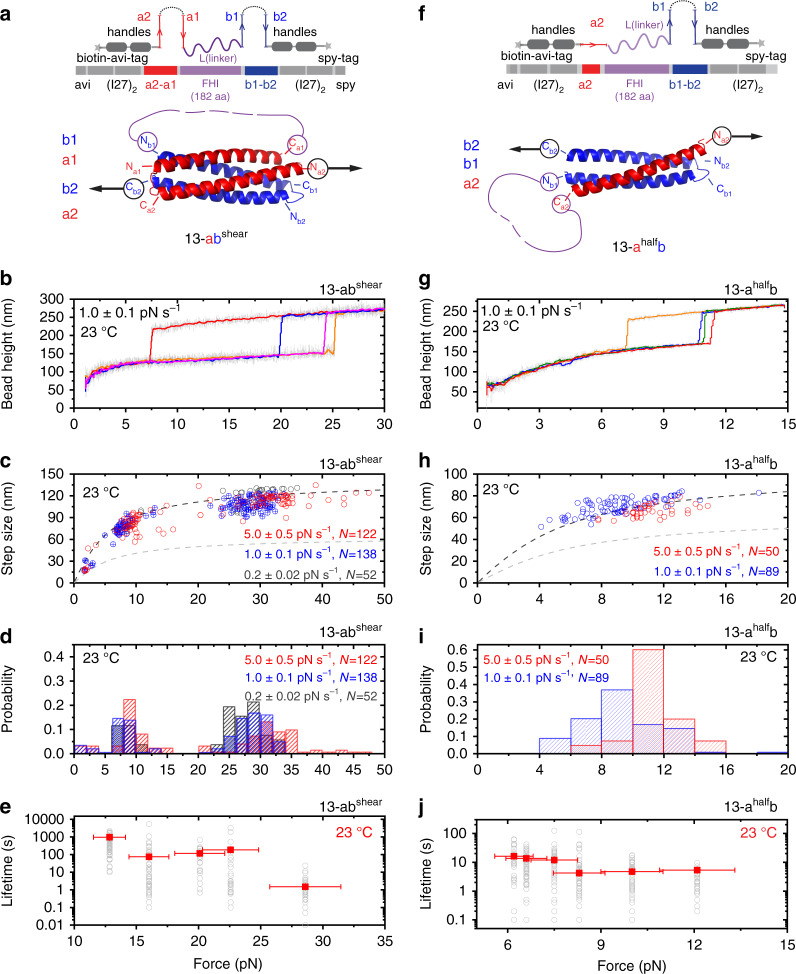
Modulating the mechanical stability of helix-bundles. **a** Top panel: design of the 13-a2a1Lb1b2 construct. Bottom panel: The expected structure of the 13-ab^shear^. **b** Four representative force–height curves of the 13-a2a1Lb1b2 construct obtained at 1.0 ± 0.1 pN s^−1^. The stepwise bead height jump indicates the force-induced rupturing (unlooping) of the 13-ab^shear^. **c** The force–step size graph of the force-dependent rupture transitions of the 13-ab^shear^ that causes unlooping of the construct. The dash curves are theoretically predicted. **d** Normalized histograms of the 13-ab^shear^ rupture forces. **e** Force-dependent lifetimes of the 13-ab^shear^. **f** Top panel: design of the 13-a2Lb1b2 construct. Bottom panel: the expected structure of the 13-a^half^b. **g** Four representative force–height curves of the single-molecule 13-a2Lb1b2 construct at 1.0 ± 0.1 pN s^−1^. The stepwise bead height jump indicates the force-induced rupturing (unlooping) of the 13-a^half^b. The force–step size graph of the force-dependent rupturing (unlooping) transitions of the 13-a^half^b. The dash curves are theoretically predicted. **i** Normalized histograms of the 13-a^half^b helix-heterotrimer rupture forces. **j** Force-dependent lifetimes of the 13-a^half^b helix-heterotrimer. For panels **b**–**e** and **g**–**j**: the experiments were performed at 23 °C. For panels **c**, **d**, **h**, and **i**, the number of data points obtained from >5 independent tethers are indicated. Force panels **e** and **j**, the solid squares represent the average lifetimes (*τ*) obtained by best-fitting of the lifetime histogram to a single-exponential decay function. The hollow gray circles represent individual lifetimes (*N* ~ 100 for each force) measured in experiments. The vertical error bars indicate the standard errors of the lifetime obtained by $\frac{\tau }{{\sqrt {N - 1} }}$. The horizontal error bars indicate 10% of relative force uncertainty resulted from the force-calibration method. For panels **b** and **g** 20-points FFT smooth (colored lines) of the raw data (gray lines) are shown. Source data are provided as a Source Data file.

Using the 13-ab^zip^ helix-heterotetramer as a starting building block, we explored the possibilities to modulate the mechanical stability of the helix-heterotetramer by manipulating the force geometry. Since molecular complexes under shear-force geometry typically have a stronger mechanical stability than that under the unzipping force geometry^54^, we modified the design of the construct so that the force is applied to the N-terminus of the a2 helix in the a component and the C-terminus of the b2 helix in the b component, leading to a shear-force geometry on the resulting helix-heterotetramer (Fig. 2a and Supplementary Fig. 1b). We refer the modified construct to as 13-a2a1Lb1b2 and the resulting helix-heterotetramer to as 13-ab^shear^.

Figure 2b shows the representative force–height curves of 13-ab^shear^ helix-heterotetramer during force-increase scans with a loading rate of 1 pN s^−1^. Figure 2c shows the force–step size graph of the force-dependent rupture transitions of the 13-ab^shear^ helix-heterotetramer at three loading rates of 0.2 pN s^−1^ (dark gray), 1 pN s^−1^ (blue) and 5 pN s^−1^ (red). The corresponding normalized distributions of the rupture forces are shown in Fig. 2d. At all three loading rates, a two-peak distribution was obtained. We reason that the mechanically weaker species could be an intermediate, partially folded structure involving only two or three helices. A possible mechanism that may lead to such intermediate is that the more extended flexible linker in the helix-heterotatramer formed by 13-ab^shear^ than that by 13-ab^zip^ could sterically slow down the complete folding process, resulting in such partially folded intermediate. The mechanically stronger species, which is expected to be a helix-heterotetramer involving four helices, is the major form, occupying ~70 ± 5% of the events.

As the mechanically stronger major species could provide a more stable mechanical anchorage, we quantified the force-dependent lifetime of the 13-ab^shear^ helix-heterotetramer at forces greater than 10 pN (Fig. 2e and Supplementary Fig. 8). The helix-heterotetramer can withstand high forces (15–30 pN) over a time scale of seconds to minutes. For comparison, over the similar time scale the 13-ab^zip^ helix-heterotetramer can only withstand 5–12 pN. Altogether, these results indicate that greater mechanical stability of the helix-heterotetramer can be achieved by changing the geometry of the applied force from unzipping geometry to shearing geometry.

We also explored the possibility of modulating the mechanical stability by changing the number of interacting helices. This was done by creating a construct that contains only three out of the original four helices: the a2 helix in the a helix-hairpin and the two helices in the b helix-hairpin. This construct is referred to as 13-a2Lb1b2 (Fig. 2f and Supplementary Fig. 1c). In this construct, the b component forms a stable helix-hairpin structure (Supplementary Fig. 2 and Supplementary Note 5). We expect that the joining of the a2 peptide to the b helix-hairpin could lead to formation of a mechanically stable helix-heterotrimer, referred to as the 13-a^half^b helix-heterotrimer, under a shear-force geometry.

From force-loading and force-clamping experiments, the three helices indeed form a stable complex, but with a weaker mechanical stability than either the 13-ab^zip^ or 13-ab^shear^ helix-heterotetramer (Fig. 2g–j and Supplementary Fig. 9). Its rupturing force distribution (Fig. 2i) is similar to the mechanically weaker species observed in Fig. 2d, consistent with the possibility that the mechanically weaker species of 13-ab^shear^ corresponds to an intermediate structure that involves three interacting helices. This result suggests that it is possible to modulate the mechanical stability via changing the number of interacting helices.

### The temperature-dependent mechanical stability

**Fig. 3:**
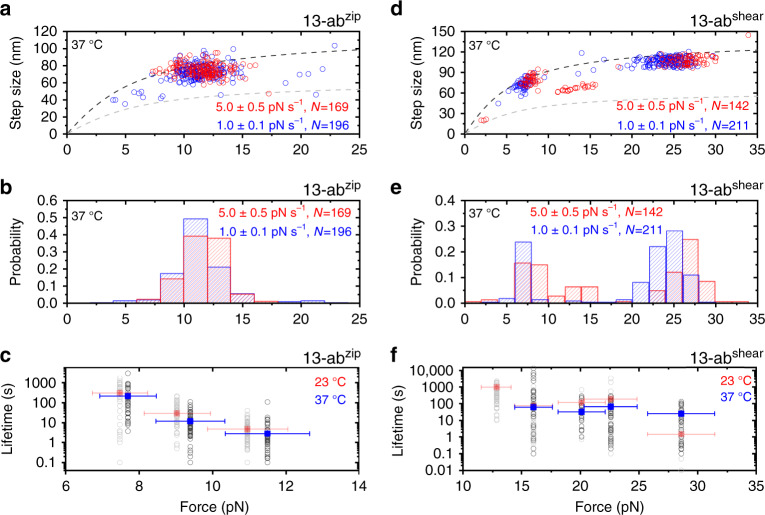
Temperature-dependent mechanical stability of helix-heterotetramers. **a** The force–step size graph of the force-dependent rupturing (unlooping) transitions of the 13-ab^zip^ obtained at 37 °C. The dash curves are theoretically predicted. **b** Normalized histograms of the 13-ab^zip^ rupture forces obtained at 37 °C. **c** Force-dependent lifetimes of the 13-ab^zip^ at 37 °C (blue) and 23 °C (red, same as Fig. 1f). **d** The force–step size graph of the force-dependent rupturing (unlooping) transitions of the 13-ab^shear^ obtained at 37 °C. The dash curves are theoretically predicted. **e** Normalized histograms of the 13-ab^shear^ rupture forces obtained at 37 °C. **f** Force-dependent lifetimes of the 13-ab^shear^ at 37 °C (blue) and 23 °C (red, same as Fig. 2e). For panels **a**, **b**, **d**, and **e**: the number of data points obtained from >5 independent tethers are indicated. For panels **c** and **f**: the solid squares represent the average lifetimes (*τ*) obtained by best-fitting of the lifetime histogram to a single-exponential decay function. The hollow gray circles represent individual lifetimes (*N* ~ 100 for each force) measured in experiments. The vertical error bars indicate the standard errors of the lifetime obtained by $\frac{\tau }{{\sqrt {N - 1} }}$. The horizontal error bars indicate 10% of relative force uncertainty resulted from the force-calibration method. Source data are provided as a Source Data file.

The ability of the helix-heterotetramers to retain their mechanical stability at higher temperature is important for its versatile applications at various temperatures. Hence, we quantified the mechanical stability of the ab^zip^ and ab^shear^ helix-heterotetramers at the human body temperature ~37 °C. For the 13-ab^zip^ and 13-ab^shear^ helix-heterotetramers, the force-loading rate-dependent rupture force distribution (Fig. 3a–b, d–e) and the force-dependent lifetimes (Fig. 3c, f and Supplementary Figs. 10 and 11) show moderate changes from the results obtained at 23 °C. Impressively, the helix-heterotetramer formed by 13-ab^shear^ retained significant mechanical stability at the highest temperature (~47 °C) tested in our experiments (Supplementary Fig. 12). These results suggest that the helix-heterotetramers still have sufficient mechanical stability at 37 °C which can be used over physiological temperature range.

### The formation of helix-heterotetramer is rapid and robust

**Fig. 4:**
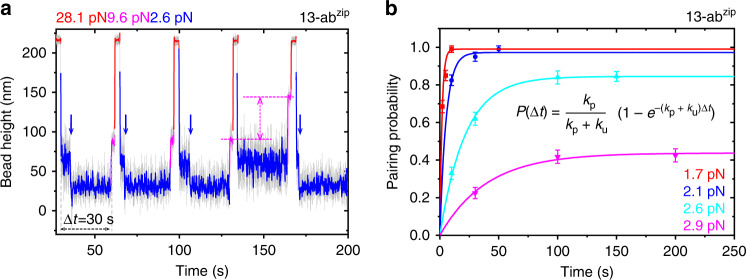
Force-dependent looping rate of helix-heterotetramer. **a** An example of the force-clamping procedure to quantify the rate of the formation of the helix-heterotetramer in the linked construct 13-a1a2Lb1b2 at various forces. After jumping from a higher force (~28.1 pN) where the construct is in the unlooped state to a lower force (~2.6 pN), the average looping probability is measured within certain waiting time (Δ*t* = 30 s) in multiple cycles. The looping transition is indicated by a stepwise bead height decrease at ~2.6 pN (blue arrows). The magenta dash lines indicate the bead height difference between looped and unlooped states at 9.6 pN (~70 nm). **b** Time-dependent looping probability of the construct, *P*(Δ*t*) at different forces indicated by different colors. The vertical error bars indicate standard errors. Colored lines represent the exponential fitting of the pairing probability ${\mathrm{P}}\left( {\Delta t} \right) = \frac{{k_p}}{{k_p + k_u}}\left( {1 - e^{ - (k_{\mathrm{p}} + k_{\mathrm{u}})\Delta t}} \right)$. Source data are provided in the Source Data file.

The ability to rapidly form the helix-heterotetramer is also important for its various applications. Hence, we quantified the rate of the helix-heterotetramer formation. Briefly, we first kept the 13-a1a2Lb1b2-construct in the unlooped state at a sufficiently high force, followed by a force jump to a lower value of 1–3 pN, and then held the construct at the lower force for a time duration of $\Delta t = 2,\;5,\;10, \ldots$ sec, to allow the potential formation of the 13-ab^zip^ helix-heterotetramer (Fig. 4a). If the 13-ab^zip^ helix-heterotetramer is formed during Δ*t*, the bead height will be shortened due to the looping of the linker (blue arrows in Fig. 4a). At forces >1 pN, the extension difference between the looped and the unlooped states is >10 nm. However, due to larger thermal motion of the bead at lower forces, the extension difference might not be visually clear enough at short duration of Δ*t* (Supplementary Fig. 13). Therefore, we added an additional force of ~9 pN to distinguish between the looped and the unlooped states of the construct. At this detecting force, the extension difference between two states is >70 nm (magenta arrow in Fig. 4a), which allows us to unambiguously determine the state of the construct prior to the force jump. By repeating the force-jumping assay over one hundred times from more than five tethers, we obtained the probability of the formation of the 13-ab^zip^ helix-heterotetramer at given lower forces over the waiting time Δ*t* (Fig. 4b).

The resulting time-dependent probability of forming the 13-ab^zip^ helix-heterotetramer at given forces show that at increased forces, the time taken for the looping probability to reach an equilibrium state increased. The looping probability can be fitted to ${\mathrm{P}}\left( {\Delta t} \right) = \frac{{k_p}}{{k_p + k_u}}\left( {1 - e^{ - (k_{\mathrm{p}} + k_{\mathrm{u}})\Delta t}} \right)$, where *k*_p_ and *k*_u_ are the rates of looping (13-ab^zip^ helix-heterotetramer formation) and unlooping (13-ab^zip^ helix-heterotetramer rupturing), respectively, which can be determined by fitting to the experimentally measured time evolution of the looping probability (Fig. 4b and Supplementary Table 1).

The values show that *k*_p_ has a strong dependence on force, deceasing over 50 folds when force increased from 1.7 pN (~0.58 s^−1^) to 2.9 pN (~0.01 s^−1^). In contrast, *k*_u_ is much less sensitive to force over the tested force range, with a value of ~0.005–0.015 s^−1^ around the force range. The strong force dependence of the looping rate is consistent with a large transition distance as a result from the looping of the flexible linker to reach the transition state. Applying the Arrhenius rate equation, a zero-force looping rate is estimated to be ~15.7 s^−1^ based on the *k*_p_(*F*) data points obtained at the four force values (Supplementary Table 1, Supplementary Notes 6–7, and Supplementary Fig. 14). Furthermore, the formation of the 13-ab^zip^ helix-heterotetramer is highly robust, as it can be formed over hundreds of cycles of looping and unlooping. Overall, these results collectively suggest that the 13-ab^zip^ helix-heterotetramer formation is rapid and robust.

To find whether other reported helix-heterotetramer systems have similar properties, we also quantified the #37 helix-heterotetramer under both unzipping (Supplementary Figs. 15a, 16–21) and shearing force (Supplementary Figs. 15b, 18, 19, 22–24) geometries. The results show that the mechanical stability of the #37 helix-heterotetramer, and its dependence on the force geometry and temperature, are similar to those of the #13 helix-heterotetramer. Therefore, the principle of modulating the mechanical stability of the complexes could be generally applied to all the helix-heteroteramers in the reported list^49^.

### Helix-heterotetramer as a mechanically stable anchorage

We have shown that the #13 and #37 helix-heterotetramers can withstand a significant range of mechanical forces over physiological temperature range. The formation of the helix-heterotetramers is also rapid and robust. These properties make them appealing candidates to be used as mechanically stable anchorage/crosslinker over physiological temperature range. We demonstrate helix-heterotetramer system’s applications as a mechanically stable anchorage in single-molecule studies of force and temperature-dependent protein unfolding/refolding dynamics and protein stability.

**Fig. 5:**
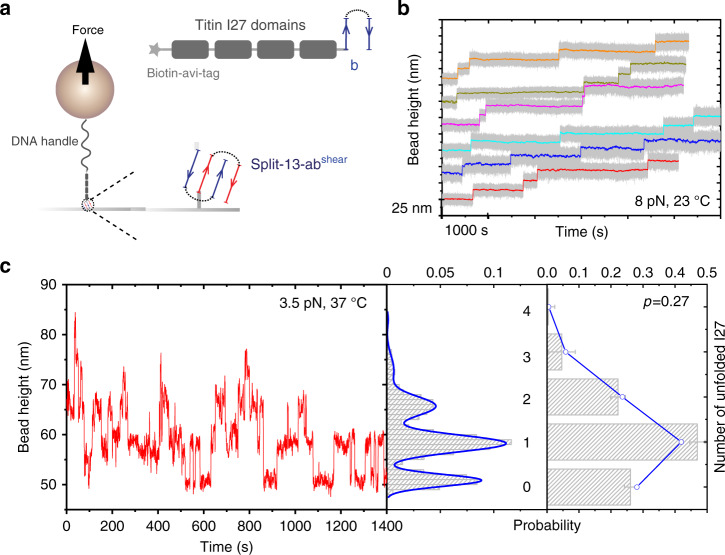
Temperature-dependent titin I27 unfolding/refolding dynamics. **a** Experimental design of utilizing the split-13-ab^shear^ helix-heterotetramer to anchor a protein construct (bio-4I27-b) that contains four repeats of titin I27 domains to coverslip surface. The I27 domains are spanned between an avi-biotin tag at N-terminus and the #13-b helix-hairpin at the C-terminus. The bio-4I27-b construct is specifically tethered to the #13-a helix-hairpin coated bottom surface via formation of split-13-ab^shear^ complex, and the N-terminus is tethered to the DNA-coated magnetic bead through biotin–streptavidin interaction (details are provided in Supplementary Note 2). **b** Six representative time traces of the bead height obtained from six independent tethers under a constant force of ~ 8 pN at 23 °C. The four stepwise extension increase events with a step size of ~15 nm indicate I27 unfolding. Colored lines are 200-points FFT smooth of the raw data (gray). **c** Left panel: a representative time trace of the bead height (20-points FFT smoothed) under a constant force of ~4 pN at 37 °C. The stepwise extension increase/decrease with step sizes of ~10 nm indicates unfolding/refolding of the I27 domains. Middle panel: the corresponding probability distributions of the bead height. Blue line is the multiple peak Gaussian fitting of the bead height probability distributions. Right panel: The bar graph shows the probability of having *n* unfolded I27 domains in the four I27 domains obtained from the bead height distribution in the middle panel. The error bar indicates the standard error, which is obtained through multiple peak Gaussian fitting of the bead height probability distributions. Blue curve indicates the fitting of the bar graph to the binomial distribution, from which the probability of the unfolded state of a single I27 domain at this force is determined to be: $p\left( F \right)\sim 0.27$ at 37 °C. Source data are provided in Source Data file.

In the first example, we used the #13 helix-heterotetramer to anchor a protein construct (bio-4I27-b) containing four repeats of titin I27 domains (Fig. 5a). In this construct, the I27 domains are spanned between an avi-biotin tag at N-terminus and the b helix-hairpin at the C-terminus. The C-terminus of bio-4I27-b construct is specifically tethered to the complementary a helix-hairpin immobilized on the bottom coverslip surface under the shear-force geometry (referred as split-13-ab^shear^, Supplementary Note 2). The biotin-tagged N-terminus is tethered to a superparamagnetic bead via a 572 bp DNA handle (Fig. 5a, left). As previously mentioned, I27 has a low unfolding rate (~10^−3^ s^−1^) within 50 pN^27,54^. On the other hand, the 13-ab^shear^ helix-heterotetramer has a comparable unfolding rate at forces <10 pN at 23 °C (Fig. 2e). Therefore, at forces within 10 pN, the long lifetime of the 13-ab^shear^ helix-heterotetramer can potentially be utilized as a mechanically stable anchorage to investigate the mechanical stability of I27.

Figure 5b shows six representative time traces of the bead height obtained from six different tethers after a force jump from <1 pN, at which all domains are folded, to a constant force of ~8 pN at 23 °C. In each time trace, four stepwise increases of the bead height are observed, corresponding to unfolding of the four I27 repeats. The step sizes are distributed around 14.9 ± 1.0 nm (mean ± standard deviation), consistent with the release of ~89 residues of I27 into a disordered polypeptide (Supplementary Note 6 and Supplementary Figs. 4 and 5). Refolding of the unfolded domains was not observed at this force, because the force is greater than the equilibrium critical force of I27 (~5.4 pN) at 23^ o^C as reported previously^27,31^. At this force, the I27 domain has very slow unfolding rate, resulting in long experimental time duration of more than one and half hour for each experiment. This example demonstrates that the long lifetime of the split-13-ab^shear^ helix-heterotetramer under shear-force geometry over physiological force range, typically a few pN, provides a specific anchoring method to support long duration of single-molecule experiments.

After unfolding, I27 domain exists in a highly disordered state that carries a larger conformational entropy^27^. Therefore, increased temperature is expected to decrease the domain stability. In order to probe the thermodynamic properties of I27, we increased the temperature to 37 °C and measured the equilibrium unfolding and folding transitions, which were used to calculate the folding energy of I27. Figure 5c is a representative time trace of the bead height obtained at ~3.5 pN showing reversible unfolding and folding transitions. From the trace, we obtained the probability distributions of bead height during dynamic unfolding and refolding of the four I27 domains. The probability of having *n* unfolded I27 domains in four independent I27 repeats, *P*_4_(*n*), can be directly read out from the bead height distribution peaks (Fig. 5c, right panel). It follows the binomial distribution: $P_4(n) = C_4^np^n(1 - p)^{4 - n}$, where $C_4^n = \frac{{4!}}{{n!\left( {4 - n} \right)!}}$ is the binomial coefficient. The single-free parameter *p* denotes the probability of an I27 domain in the unfolded state at this force.

Fitting of the binomial distribution to the normalized probability of the number of unfolded I27 repeats, we determined that *p*(*F*) ~ 0.27 at 3.5 pN at 37^ o^C (Fig. 5c). The value of *p*(*F*) is related to the force-dependent free energy difference between the unfolded and folded states, Δ*G*(*F*), by $\Delta G\left( F \right) = \Delta G_0 + \Delta \phi \left( F \right) = - k_{\mathrm{B}}T{\mathrm{ln}}\frac{{1 - p(F)}}{{p(F)}}$. Here, $\Delta G_0$ is the zero-force folding energy of the I27 domain, $\Delta \phi \left( F \right) = \mathop {\smallint }\limits_0^F (x_{\mathrm{u}}(f) - x_{\mathrm{f}}(f)){\mathrm{d}}f$ is the force-dependent conformational free energy difference between the unfolded state and the folded state^27,31^. Using the equation and based on the measured *p*(*F*) and force-extension curves of I27 in the folded and unfolded states (Supplementary Note 6), we obtained $\Delta G_0 = - 4.9 \pm 0.4\;k_{\mathrm{B}}T$. The critical force $F_c\sim 3.9$ pN at which the unfolded and folded states have equal probabilities, is also obtained by $\Delta G_0 + \Delta \phi \left( {F_c} \right) = 0$. Comparing these values with those obtained at 23 °C^27,31^ (Table 1), it clearly shows that when temperature increases from 23 to 37 °C, $\Delta G_0$ of I27 increases significantly by more than 3 *k*_B_T (i.e., thermodynamic stability decreases) and correspondingly the equilibrium critical force decreases by ~1.5 pN.

**Table 1:** Thermal stability of the **α**-actinin 1 SR4 and titin I27 domains.

	*α-*Actinin 1 SR4				Titin I27	
*F* (pN)	5.7 ± 0.57	5.7 ± 0.57	5.7 ± 0.57	5.7 ± 0.57	4.5 ± 0.45	3.5 ± 0.35
*T* (^o^C)	23	27	29	31	23	37
p(*F*)	0.091 ± 0.002	0.283 ± 0.004	0.531 ± 0.005	0.722 ± 0.008	0.131 ± 0.004	0.272 ± 0.004
$\Delta \phi (F)$ (*K*_B_*T*)	10.90 ± 2.03	10.38 ± 2.01	10.31 ± 1.99	10.15 ± 1.96	6.44 ± 1.1	3.95 ± 0.7
$\Delta G(F)$ (*K*_B_*T*)	−2.30 ± 0.02	−0.93 ± 0.02	0.12 ± ± 0.02	0.95 ± 0.04	−1.89 ± 0.05	−0.98 ± 0.04
${\mathrm{\Delta }}G_0$ (*K*_B_*T*)	−13.2 ± 2.0	−11.3 ± 2.0	−10.2 ± 2.0	−9.2 ± 2.0	−8.3 ± 1.0	−4.9 ± 0.7
*F*_*c*_ (pN)	6.3 ± 0.5	6.0 ± 0.6	5.7 ± 0.6	5.4 ± 0.6	5.4 ± 0.4	3.9 ± 0.3

For *α*-actinin 1 SR4 domain, experiments were performed at four different temperatures (23, 26, 29, and 31 °C) at a constant force of 5.7 pN. For titin I27 domain, experiments were performed at 37 °C at a constant force of 3.5 pN. The data for titin I27 at 23 °C at a constant force of 4.5 pN were taken from previous publications^27,31^ for comparison. The error bars (s.t.d.) for *p*(*F*) are obtained by bootstrap analysis; for ΔΦ(*F*) are obtained by error propagation from the 10% of relative uncertainty in force-calibration (“Methods”); for Δ*G*(*F*) are obtained by error propagation from the error bars of *p*(*F*); for Δ*G*_0_ are obtained by error propagation from the error bars of ΔΦ(*F*) and Δ*G*(*F*); for *F*_*c*_ are obtained by error propagation from the error bars of Δ*G*_0_. Source data are provided as a Source Data file.

**Fig. 6:**
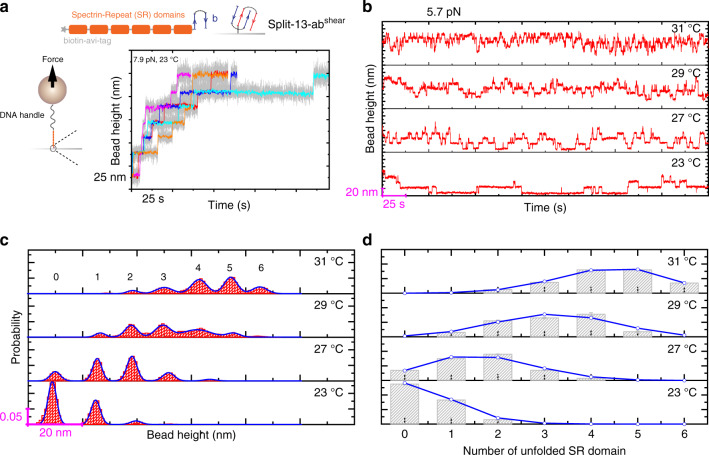
Temperature-dependent α-actinin SR4 unfolding/refolding dynamics. **a** Top panel: experimental design of utilizing the split-13-ab^shear^ to anchor a protein construct (bio-6SR-b) that contains six repeats of the fourth spectrin-repeat domain to glass surface. The six SR domains are spanned between an avi-biotin tag at N-terminus and the #13-b helix-hairpin at the C-terminus. The bio-6SR-b construct is specifically tethered to the #13-a helix-hairpin coated bottom surface via formation of split-13-ab^shear^ complex, and the N-terminus is tethered to the DNA-coated magnetic bead through biotin–streptavidin interaction (Supplementary Note 2). Bottom panel: five representative time traces of the bead height obtained from five independent tethers under a constant force of ~7.9 pN at 23 °C. The six stepwise extension increase events with a step size of ~25 nm in each time trace indicate SR unfolding. Colored lines are 20-points FFT smooth of the raw data (gray). **b** Representative time traces of the bead height (20-points FFT smoothed) under a constant force of ~5.7 pN at multiple temperatures (23, 27, 29, and 31 °C). The stepwise extension increases and decreases with step sizes of ~18 nm indicate unfolding/refolding of the SR domains. **c** The resulting probability distributions of the bead height. The numbers of unfolded spectrin-repeat domains at corresponding bead height are indicated. Blue line is the multiple peak Gaussian fitting of the bead height probability distributions. **d** The bar graph shows the probability of having *n* unfolded SR domains in the six SR domains obtained from the bead height distribution in **c**. The error bar indicates the standard error, which are obtained through multiple peak Gaussian fitting of the bead height probability distributions. Blue curve indicates the fitting of the bar graph to the binomial distribution. Source data are provided in Source Data file.

In the above example, the I27 domain has a typical β-sheet structure. In order to probe the thermodynamic properties of protein domains with different ternary structures, such as α-helix bundles, we created another protein construct (bio-6SR-b) that contains six repeats of the fourth α-actinin spectrin-repeat (SR) domain^55^, spanned between an avi-biotin tag at N-terminus and the b helix-hairpin of the #13 helix-heterotetramer system at the C-terminus. Each SR domain is formed with three α-helices bundled together. Figure 6a shows representative time traces of the bead height obtained from six independent tethers, each containing six stepwise domain unfolding steps. The step sizes are distributed around ~17.9 ± 1.0 nm, consistent with the release of ~104 residues of SR4 into a disordered polypeptide (Supplementary Note 6 and Supplementary Figs. 4 and 5). Using similar approach to quantify the temperature-dependent thermal stability of I27, we quantified the temperature-dependent thermal stability of the spectrin-repeat based on equilibrium two-state transitions over a temperature range from 23 °C to 31 °C (Fig. 6b–d and Table 1), and revealed that the stability of the spectrin-repeat is negatively influenced by increased temperatures.

## Discussion

In summary, by mechanically characterizing two engineered helix-heterotetramers reported in ref. ^49^ using single-molecule construct assays, we show that their mechanical stabilities are retained over physiological temperature range. Their mechanical stability can be modulated by changing the force geometry and by changing the number of interacting helices. As the two helix-heterotetramers investigated in this study were randomly chosen from the list of the six biochemically characterized orthogonal helix-heterotetramers^49^, the retained mechanical over physiological temperature range could be a shared property of several helix-heterotetramers of the reported list^49^. The principle can be extended to other reported helix-heterotetramers, highlighting the potential for development of a variety of helix-heterotetramers with different mechanical stability for specific linking of protein domains.

Our results demonstrate that, through modulating the force geometry, the force-dependent lifetime of the two tested helix-heterotetramers can be tuned to cover the typical physiological force range over typical physiological time scale^1–7^. The mechanical stability of the helix-heterotetramers is greater under shearing force geometry than under unzipping force geometry, consistent with recent theoretical studies^40,54^. Impressively, despite their small size (~150 a.a. in the complex), a single helix-heterotetramer under shear-force geometry can survive for minutes at forces below 20 pN, which reaches the mechanical strength of antibody/antigen complexes that are much larger in size. We also show that mechanical stability is positively dependent on the number of interacting helices, suggesting another potential route to tune the mechanical stability via increasing the range of possible interacting helices. Sequence modifications of the helix-heterotetramers, which were not explored in this study, provide another possibility. However, for the helix-heterotetramers developed in ref. ^49^, sequence modifications may be unwantedly destabilizing considering that these helix-heterotetramers have been optimized for high stability^49^. It is important to note that our conclusions are based on the two tested helix-heterotetramers. Therefore, a more comprehensive understanding of how the mechanical stability of helix-heterotetramers depend on force geometry, the number of interacting helices and the sequence modifications warrants future studies.

One exciting feature is that the two helix-heterotetramers investigated in this study retain significant mechanical stability at 23 and 37 °C, which spans the typical physiological temperature range. The temperature dependence of the mechanical stability of the two helix-heterotetramers is related to the molecular details of the interactions on the transition pathways during mechanical rupture of the complexes. The very high thermodynamic stability of the two tested helix-heterotetramers characterized by melting temperatures >95 °C^49^ suggest that they contain a large fraction of endothermic hydrophobic interfaces distributed throughout the helix-heterotetramers. Hence, the moderate influence of the temperature on the mechanical stability of the two tested helix-heterotetramers when temperature increases from 23 to 37 °C can be explained by the presence of a significant fraction of the endothermic hydrophobic interactions on the transition pathways. Based on this principle, we propose that the key to decrease the temperature sensitivity of the mechanical stability of a protein complex is to design a transition pathway containing significant amount of endothermic hydrophobic interactions.

The helix-heterotetramers that retain significant mechanical stability over physiological temperature range is a highly advantageous property for their use as force-bearing adapter molecules in a broad range of applications. In this study, we demonstrated the application of the heterotetramer under shearing force geometry as a mechanically stable anchorage to investigate the temperature-dependent stability of two different types of protein domains (titin I27 and α-actinin spectrin-repeat) using magnetic-tweezers. We probed the equilibrium unfolding and folding transitions of these protein domains at different temperatures from 23 to 37 °C, from which we determined their thermal stability (i.e., ${\mathrm{\Delta }}G_0$) at each temperature. Our results show that for these protein domains, their thermal stability decreases as temperature increases, suggesting that the folding of these domains is overall an exothermic reaction involving predominantly enthalpy driven processes of folding. This approach can be broadly applied to investigate other protein domains to obtain insights into the physical nature of the folding processes.

Besides serving as a mechanically stable molecular anchorage for single-molecule manipulation studies, the helix-heterotetramers have many other potential applications. The specific interaction between a and b components enable their use as mechanically stable molecular crosslinkers, as they can be fused to respective target molecules (Supplementary Fig. 25). The orthogonality and the potential programmability of the multiple sets of the complementary a and b helix-pairs promise a wide scope of potential applications in developing functional materials. For example, the helix-heterotetramers are promising reversible crosslinkers of biopolymers such as polyprotein peptides or nucleic acids for engineering self-healing hydrogels^15,21,22^ or other actin/microtubule-based functional materials^56–59^ with variable mechanical properties over physiological temperature range.

The helix-heterotetramers have broad applications in mechanotransduction studies, serving as a specific building block for various specific mechanical linkages. The forces transmitted on force-transmission supramolecular linkages in the cells are in the order of a few pN^1,3,4,6^. At the cell-extracellular matrix and cell–cell adhesions, the forces transmitted on single molecules are also in similar range^2,5,7^. In addition, numerous known mechanosensing domains in different force-bearing proteins, such as talin, α-actenin and dystrophin, have been shown being unfolded by forces from a few pN to 30 pN at physiologically relevant loading rates^28,55,60^. Owing to the typical low loading rates exerted to force-transmission proteins by cells^61^, forces generated from live cells could unfold many protein domains, including the ones with high mechanical stability^62–64^. Therefore, the mechanical stability of these helix-heterotetramers is able to support studies of a wide force range of important mechanosensing proteins and mechanotransduction processes in live cells. For example, by genetically linking the complementary a and b components with actin binding domains (ABD), one can build artificial actin crosslinkers to organize actin filaments in many different ways. The crosslinking strength of such artificial actin crosslinkers can be tuned by modulating the stability of the helix-heterotetramers and by using different ABDs derived from different actin binding proteins. The length and the flexibility of the artificial actin crosslinkers can also be modulated by inserting protein spacers between the a or b component and ABDs. The highly variable design of such artificial actin crosslinkers may replace or compete with endogenous actin crosslinkers such as filamin A and α-actinin to modulate cell behaviors^65–67^.

## Methods

### Plasmids preparation and protein expression

The DNA fragments encoding a1, a2, b1, b2 helices, the long flexible linker (FH1 domain (residues 582–764 of DIAP1_HUMAN)), the four repeats of titin I27 domains (4I27), and the six repeats of the fourth α-actinin spectrin-repeat domains (6SR), as well as the avi-tag (GLNDIFEAQKIEWHE) and spy-tag(AHIVMVDAYKPTK)^68^ were synthesized by GeneArt/gBlock. The corresponding DNA fragments were then sub-cloned into expression vector pET151. Details of plasmids preparation, sequence information, and protein expression are provided in Supplementary Note 1.

### Single-molecule manipulation and data analysis

All single-molecule stretching experiments were performed using a vertical magnetic-tweezer setup^50,52,69^. The channel was combined with a disturbance-free, rapid solution-exchange method^51^ to avoid flow-drag during flow exchange. Experiments were performed in standard solution containing: 1x PBS, 1% BSA, 2 mM DTT, 10 mM sodium l-ascorbate at multiple temperatures (23, 27, 29, 31, 37, and 47 °C). The temperature was controlled by an objective heating system (Bioptechs). The details of sample preparation for single-molecule manipulation experiments are provided in Supplementary Note 2. The force-calibration of the magnetic-tweezer setup has a 10% uncertainty due to the heterogeneity of the diameter of paramagnetic beads^69^. Details of theoretical models of the force-extension curves of the folded/unfolded protein domain/complex and the force-dependent transition rates are provided in Supplementary Note 6 and Note 7, respectively.

### Reporting summary

Further information on research design is available in the Nature Research Reporting Summary linked to this article.

## Supplementary information

Supplementary Information

Supplementary Data 1

Reporting Summary

## Data Availability

Data supporting the findings of this manuscript are available from the corresponding authors upon reasonable request. A reporting summary for this Article is available as a Supplementary Information file. Source data are provided with this paper.

## Source data

Source Data
