# Supplementary material for: Modulating mechanical stability of heterodimerization between engineered orthogonal helical domains
Source: Nat Commun. 2020 Sep 8;11:4476. doi: 10.1038/s41467-020-18323-w (PMC7479118; doi:10.1038/s41467-020-18323-w)
Supplement: Supplementary file 1 — Supplementary Information [file 41467_2020_18323_MOESM1_ESM.pdf]

Supplementary Information for

Modulating Mechanical Stability of Heterodimerization Between Engineered Orthogonal Helical Domains

Yu et al.

Supplementary Figures

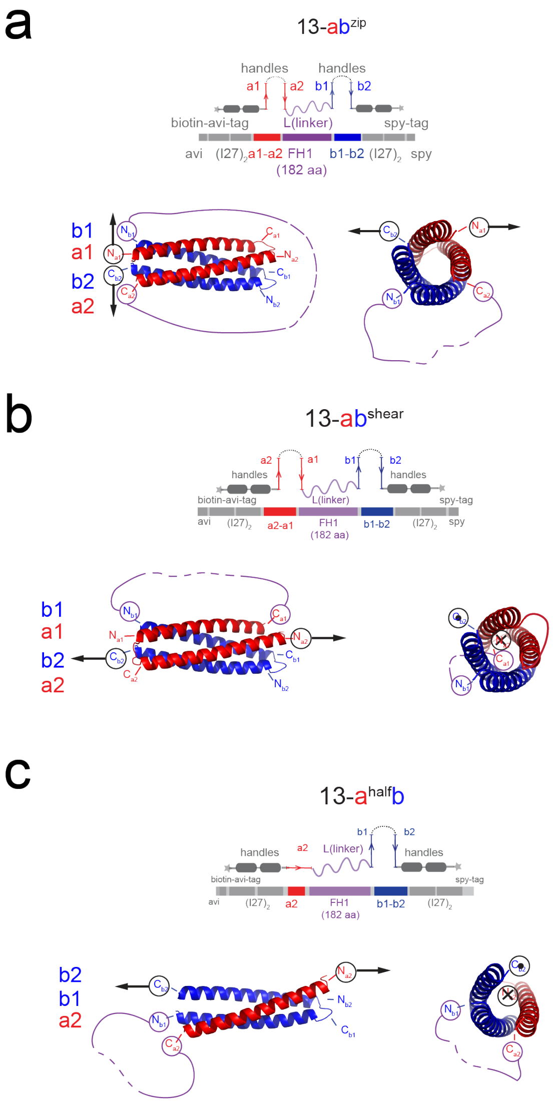

Supplementary Figure 1. The design and structure of 13-ab^{zip}, 13-ab^{shear}, and 13-a^{half}b helix bundles in side and front views. (a). The structure of 13-ab^{zip}. Top panel: single-molecule construct of 13-ab^{zip}. Bottom panel: side and front view of 13-ab^{zip} helix bundle. **(b).** The structure of 13-ab^{shear}. Top panel: single-molecule construct of 13-ab^{shear}. Bottom panel: side and front view of 13-ab^{shear} helix bundle. **(c).** The expected structure of a^{half}b. Top panel: single-molecule construct of 13-a^{half}b. Bottom panel: side and front view of the

expected 13-a^{half}b helix bundle. The a^{half}b is obtained by removing the a1 helix from the original structure of the helix-heterotetramer. For panels **a-c**: Black circles indicate the force-attaching points, purple circles indicate the attachment points of the FH1 linker. The black arrows indicate the force direction. The black dot in the black circle shows the direction of force pointing outward to the screen, the black cross in the black circle shows the direction force pointing inward into the screen.

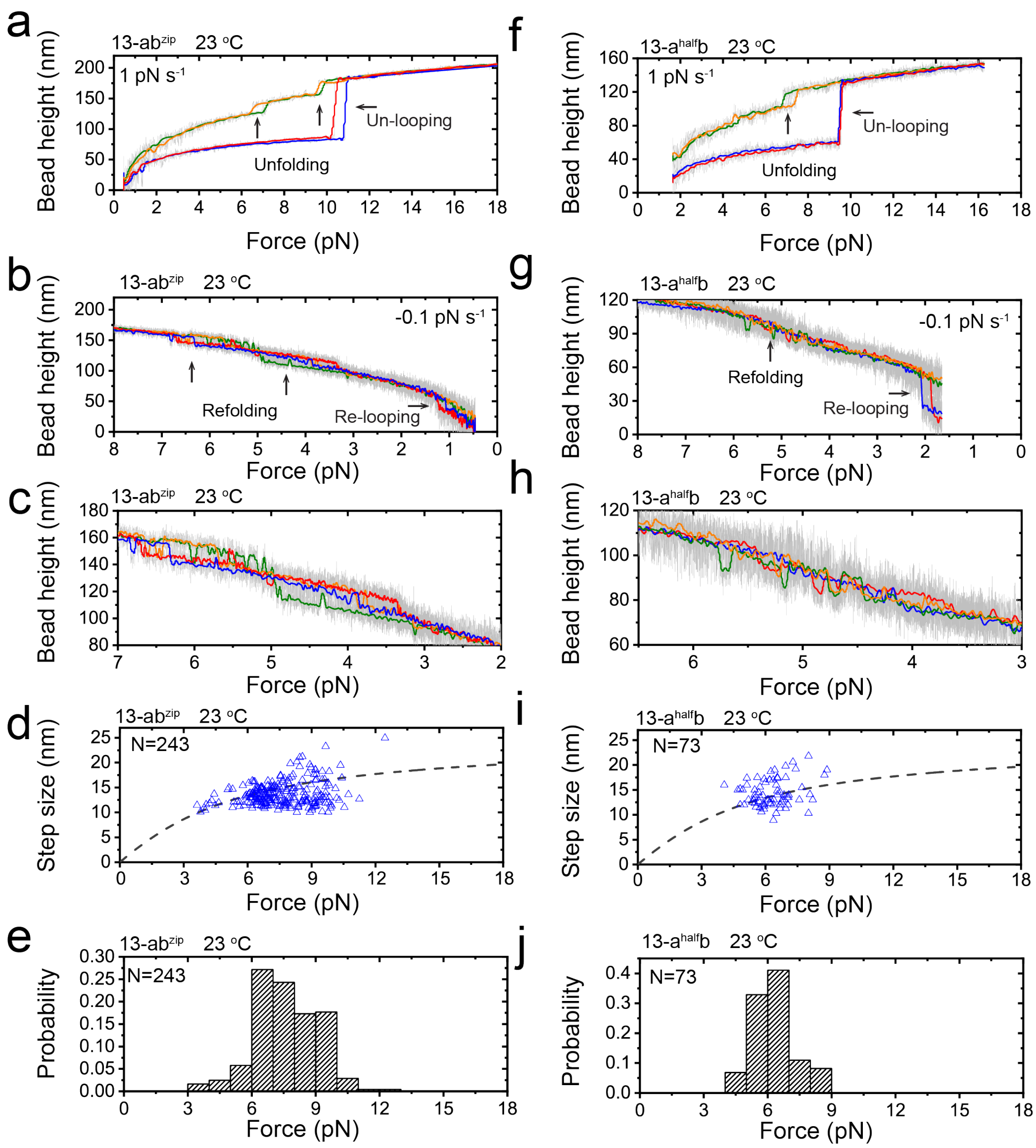

Supplementary Figure 2. Mechanical stability of the a and b helix hairpins in the 13-a^{half}b and 13-ab^{zip} constructs. (a). Four representative force-bead height curves of the 13-ab^{zip} construct during force-increase scans started from the unlooped state (green and orange curves) and looped state (red and blue curves) with a force loading rate of 1 pN s^{-1}. **(b).** Four force-bead height curves of the 13-ab^{zip} construct recorded in force-decrease scans with a force loading rate of -0.1 pN s^{-1} after the force-increase scans shown in panel **a**. **(c).** is a zoom-in of panel **b** in the force range of 2-7 pN**.** For panels **a-c**: The color lines show a 20-FFT smooth of the raw data (grey). The vertical arrows indicate the unfolding/refolding events, and the horizontal arrows indicate the unlooping/relooping events. **(d).** The force-step size graph of the force-dependent unfolding of the a and b helix hairpins of the 13-ab^{zip} construct during force-increase scans at 1 pN s^{-1}. The number of data points obtained from >5 different molecules is indicated in the panel. The dashed curves are the predicted force-step size curve of the a and b helix hairpins unfolding transition both assuming the released 76 a.a. residues exist in a disordered peptide polymer chain conformation (Supplementary Note 6, Supplementary Figures 4&5). **(e).** Normalized histogram of the unfolding forces of the a and b helix hairpins in the 13-ab^{zip} construct obtained at 1 pN s^{-1}. **(f).** Four representative force-bead height curves of the 13-a^{half}b construct started from the unlooped state (green and orange curves) and looped state (red and blue curves) with a loading rate of 1 pN s^{-1}. **(g).** Four force-bead height curves of the 13-a^{half}b

construct recorded in force-decrease scans at a loading rate of -0.1 pN s^{-1} after the force-increase scans in the panel **f**. **(h).** is a zoom-in of panel **g** in the force range of 3-6.5 pN**.** For panels **f-h**: The color lines show a 20-FFT smooth of the raw data (grey). The vertical arrows indicate the un-folding/re-folding events, and the horizontal arrows indicate the unlooping/relooping events. **(i).** The force-step size graph of the force-dependent unfolding of the b helix hairpin of the 13-a^{half}b construct during force-increase scan with a loading rate of 1 pN s^{-1}. The number of data points obtained from >5 different molecules is indicated in the panel. The dashed curve is the predicted force-step size curve of the b helix hairpin unfolding transition assuming the released 76 a.a. exists in a disordered peptide polymer chain conformation (Supplementary Note 6, Supplementary Figures 4&5). **(j).** Normalized histogram of the unfolding forces of the b helix hairpin in the 13-a^{half}b construct obtained at 1 pN s^{-1}. Source data are provided as a Source Data file.

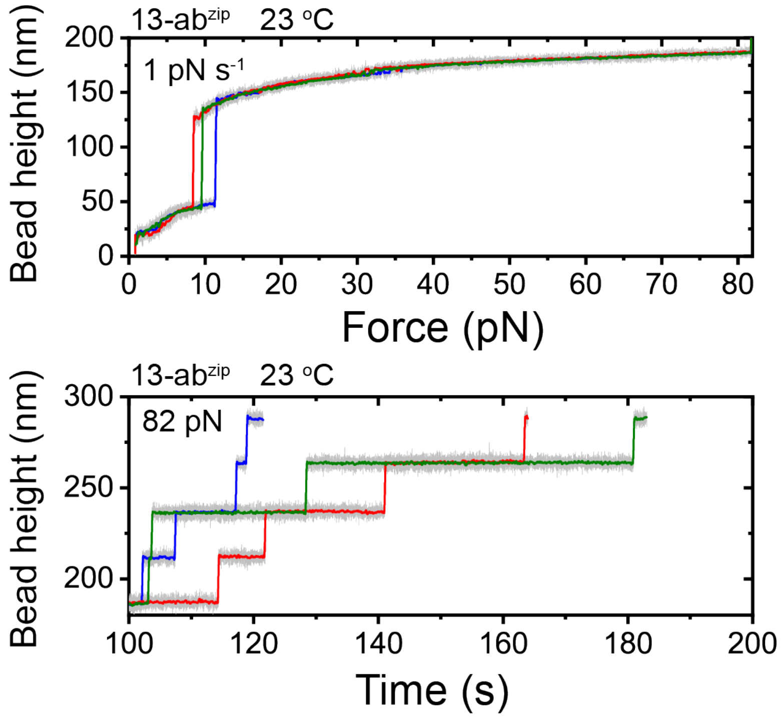

Supplementary Figure 3. Differential responses of 13-ab^{zip} helix-heterotetramer and I27 domain to force loading. top panel: Three representative force-height curves of a 13-ab^{zip} construct during force-increase scans from ~ 1 pN to ~82 pN with a loading rate of 1 pN s^{-1}. Rupturing of the ab^{zip} complex occurred at ~ 10 pN, indicated by a large stepwise height jump in each trace. Bottom panel: the same 13-ab^{zip} molecule held at ~82 pN after a force-increase scans, until all the four repeats of I27 domains were unfolded, indicated by four stepwise height jumps in each trace. The color lines show 20-FFT smooth of the raw data (grey). Source data are provided as a Source Data file.

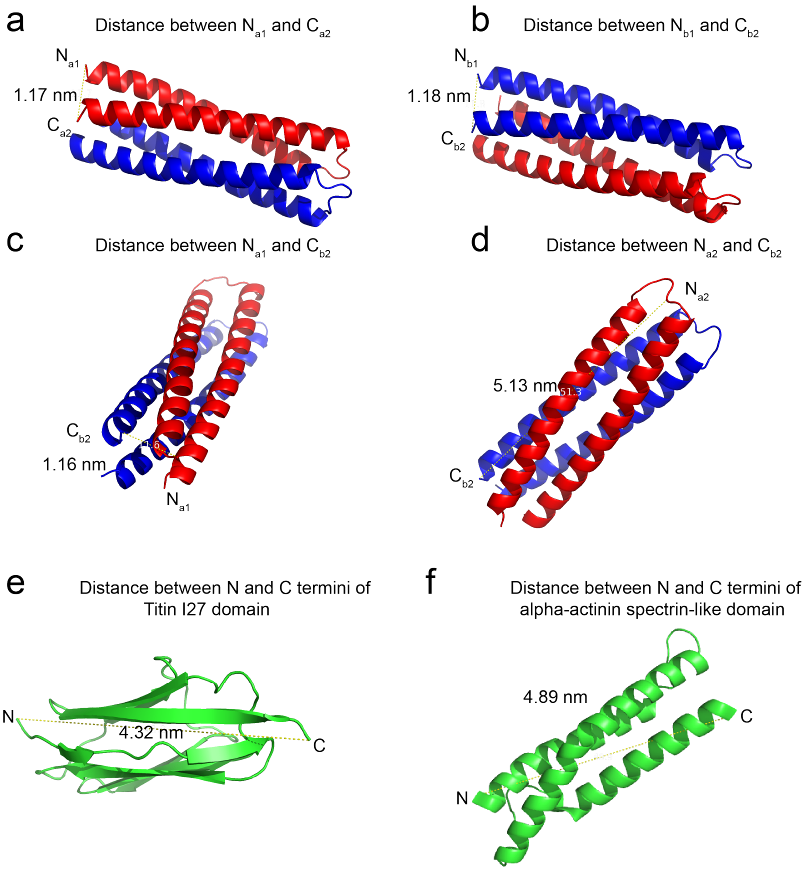

Supplementary Figure 4. The rigid body length b_0 for the folded domains.

The b_0 for #13-a helix hairpin **(a),** the #13-b helix hairpin **(b),** the 13-ab^{zip} complex **(c),** the 13-ab^{shear} complex **(d),** the titin I27 domain (PDB: 1tit) **(e),** and the alpha-actinin fourth spectrin-repeat domain (PDB: 4D1E) **(f),** are measured from the corresponding folded structures in Pymol and indicated on the figure panels.

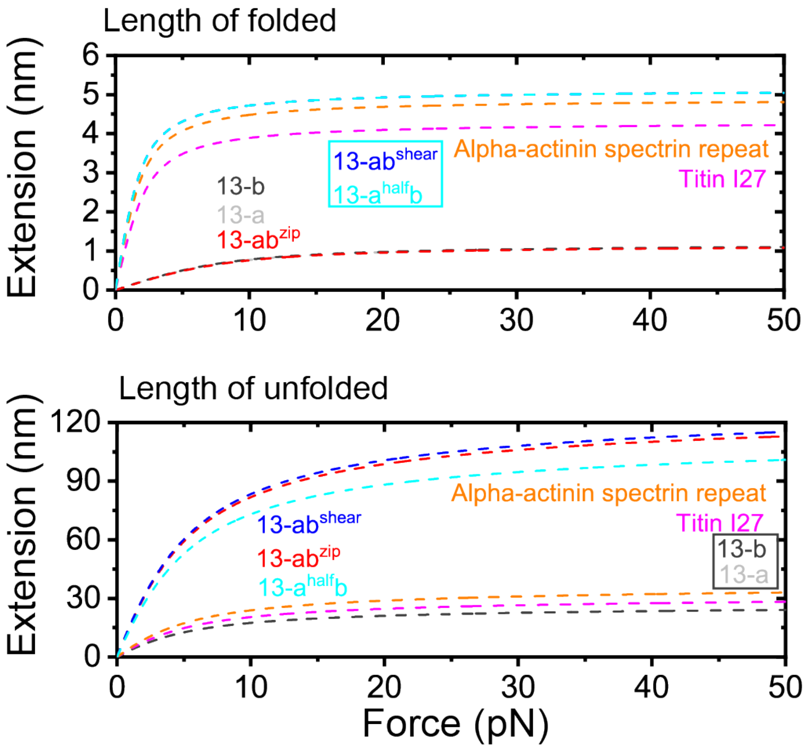

Supplementary Figure 5. Theoretical force-extension curves of the protein domains or complexes. Top panel: theoretical force-extension curve of the folded 13-a helix hairpin (grey), 13-b helix hairpin (dark grey), 13-ab^{zip} helix bundle (red), 13-ab^{shear} helix bundle (blue), 13-a^{half}b helix bundle (light blue), titin I27(magenta), and alpha-actinin spectrin-repeat domain (orange). The folded domain was considered as a single chain rigid body rotation and calculated based on free-joint chain model. The curves of 13-ab^{shear} and 13^{half}b helix bundles are overlapped in the panel. Bottom panel: force-extension curve of the unfolded 13-a helix bundle (grey), 13-b helix bundle (dark grey), 13-ab^{zip} helix bundle (red), 13-ab^{shear} helix bundle (blue), 13-a^{half}b helix bundle (light blue), titin I27(megenta), and alpha-actinin spetrin-repeat domain (orange), calculated based on an assumption that the unfolded conformation of the domain exists as an unstructured flexible peptide chain, and calculated based

on worm-like chain model. More details of the polymer models can be found in Supplementary Note 6. Source data are provided as a Source Data file.

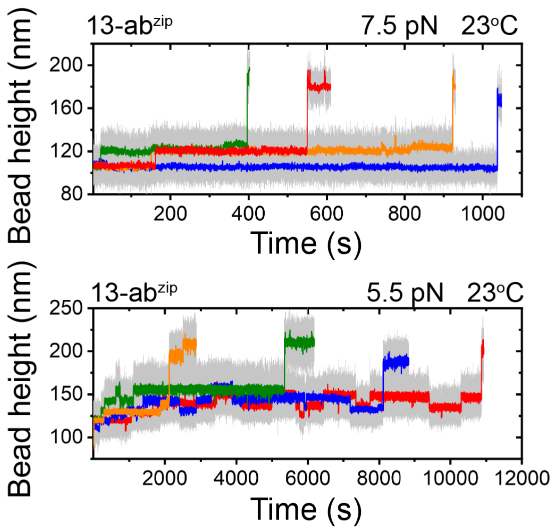

Supplementary Figure 6. Examples of the time traces of the bead height of the 13-ab^{zip} construct at constant forces. The traces started from the looped state of the molecules and ended after the unlooping transition which is indicated by a big stepwise height-increase with a size of ~ 80 nm (7.5 pN) or ~70 nm (5.5 pN). The unfolding of the I27 domains in the construct may occur before or after the unlooping transition, indicated by much smaller stepwise height increases or decreases with a size of 15-20 nm. The color lines show 20-FFT smooth of the raw data (grey). Source data are provided as a Source Data file.

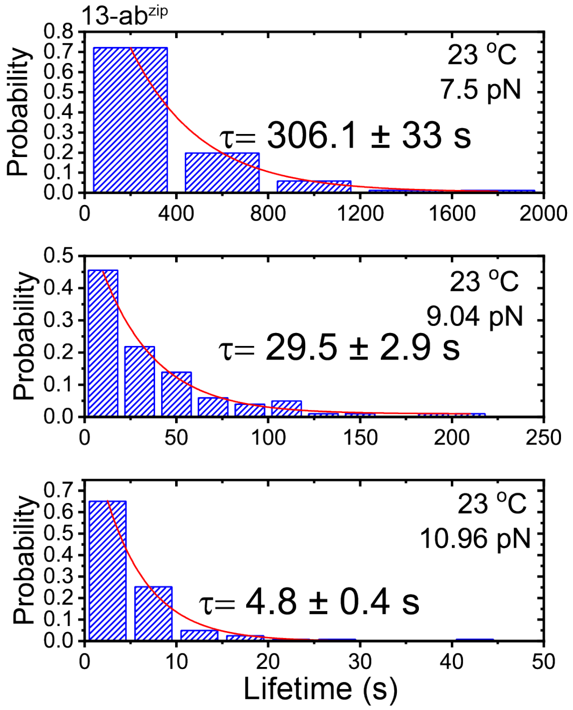

Supplementary Figure 7. Lifetime distribution of the 13-ab^{zip} complex at different forces, at 23 °C. The red curve is fitted curve of exponential decay function to the normalized lifetime distribution. The forces, fitted mean, and standard error are indicated in each panel. The number of data points obtained at 7.5 pN, 9.04 pN, and 10.96 pN are 86, 101, and 123, respectively. Source data are provided as a Source Data file.

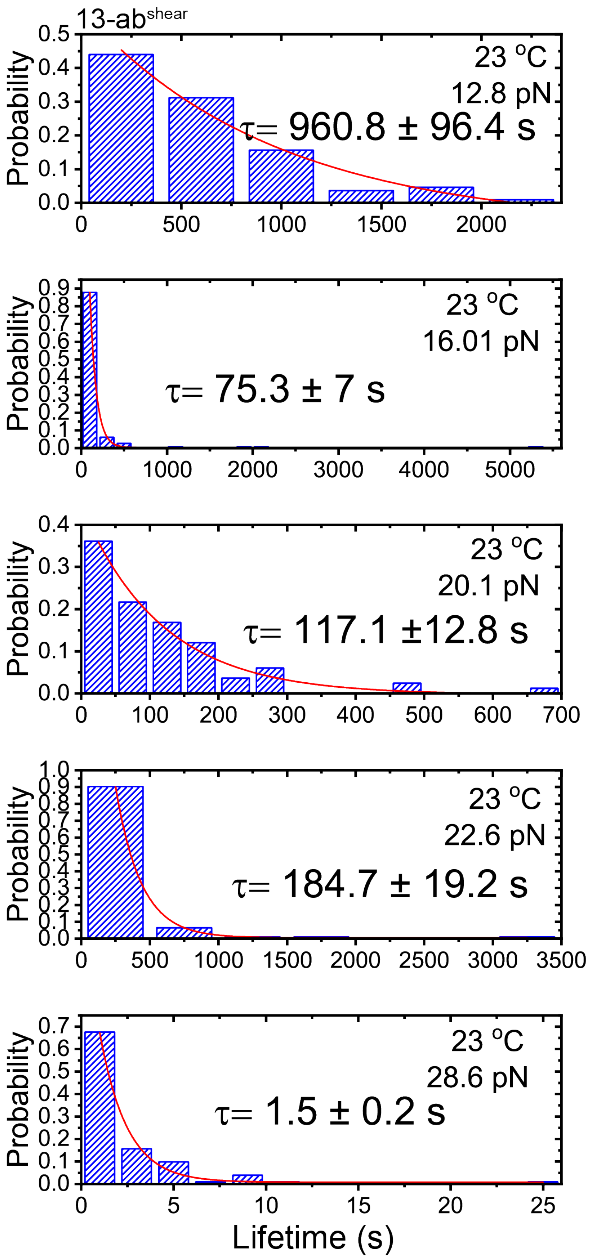

Supplementary Figure 8. Lifetime distribution of the 13-ab^{shear} at different forces, at 23 °C. The red curve is fitted curve of exponential decay function to the normalized lifetime distribution. The forces, fitted mean, and standard error are indicated in each panel. The number of data points obtained at 12.8 pN, 16.01 pN, 20.1 pN, 22.6 pN and 28.6 pN are 109, 115, 83, 92 and 102, respectively. Source data are provided as a Source Data file.

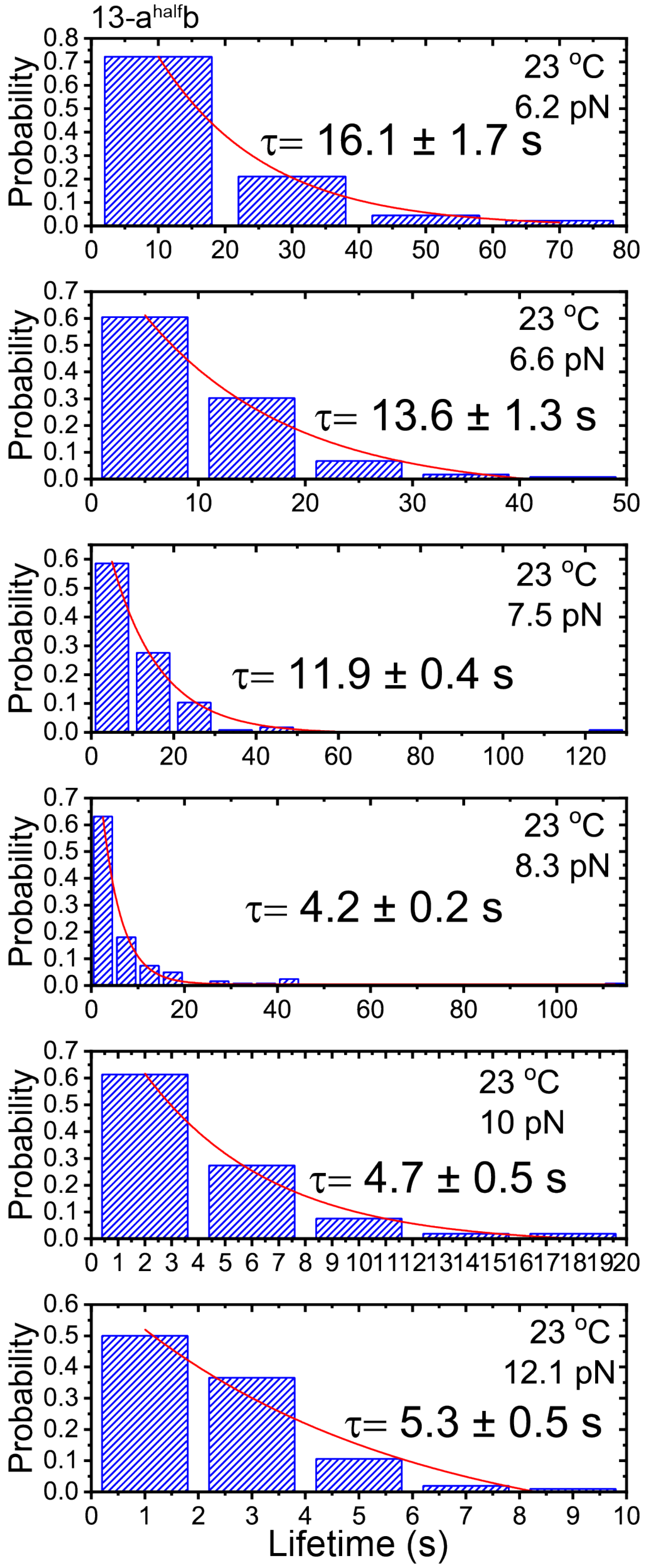

Supplementary Figure 9. Lifetime distribution of the 13-a^{half}b at different forces, at 23 °C. The red curve is fitted curve of exponential decay function to

the normalized lifetime distribution. The forces, fitted mean, and standard error are indicated in each panel. The number of data points obtained at 6.2 pN, 6.6 pN, 7.5 pN, 8.3 pN, 10 pN, and 12.1 pN are 90, 119, 116, 122, 106, and 104, respectively. Source data are provided as a Source Data file.

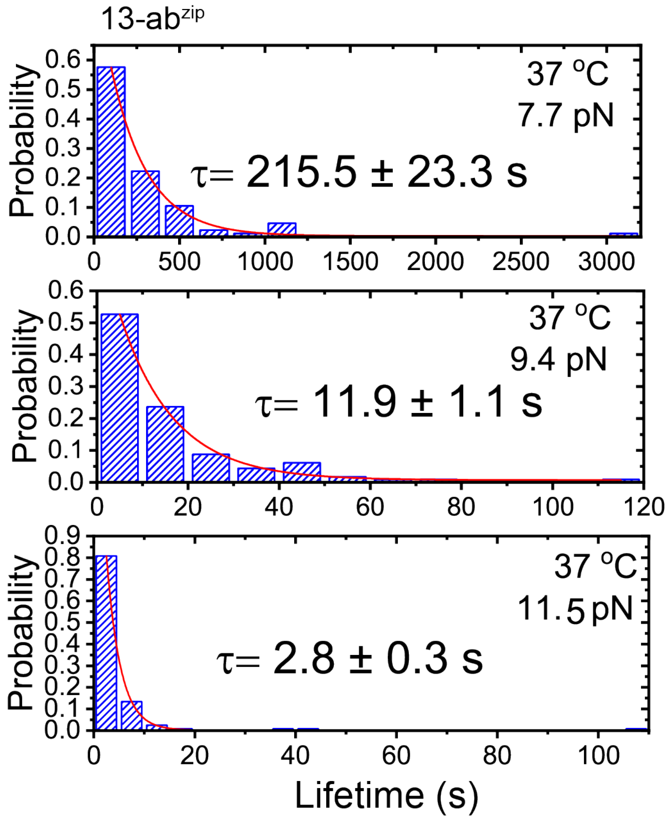

Supplementary Figure 10. Lifetime distribution of the 13-ab^{zip} at different forces, at 37 °C. The red curve is fitted curve of exponential decay function to the normalized lifetime distribution. The forces, fitted mean, and standard error are indicated in each panel. The number of data points obtained at 7.7 pN, 9.4 pN, and 11.2 pN are 85, 114, and 120, respectively. Source data are provided as a Source Data file.

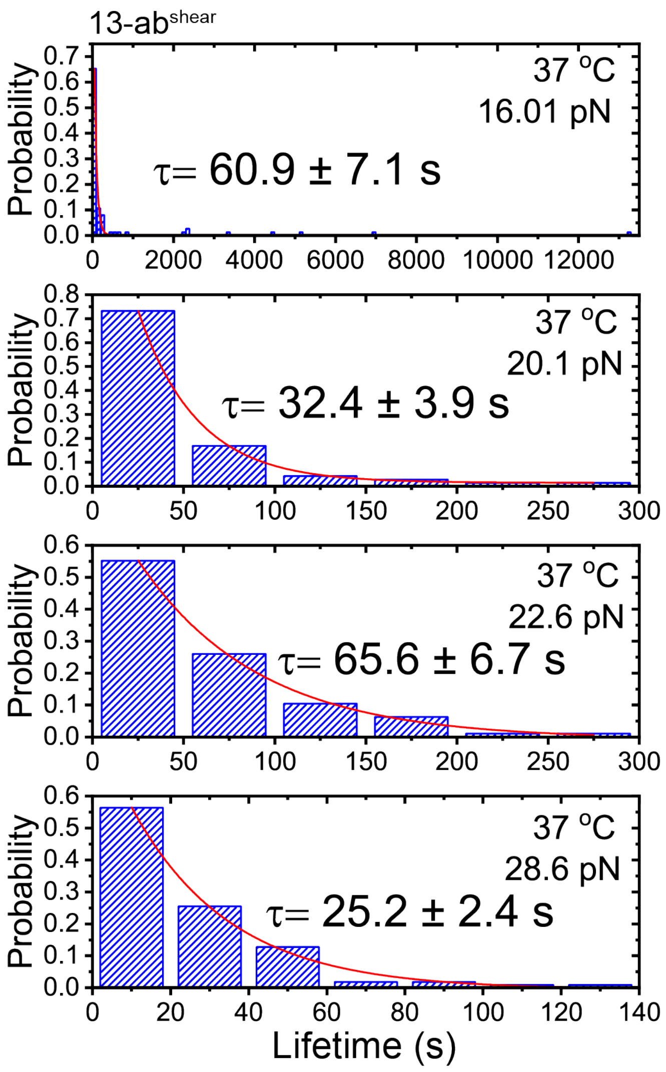

Supplementary Figure 11. Lifetime distribution of the 13-ab^{shear} at different forces, at 37 °C. The red curve is fitted curve of exponential decay function to the normalized lifetime distribution. The forces, fitted mean, and standard error are indicated in each panel. The number of data points obtained at 16.01 pN, 20.1 pN, 22.6 pN, and 28.6 pN are 75, 71, 96, and 110, respectively. Source data are provided as a Source Data file.

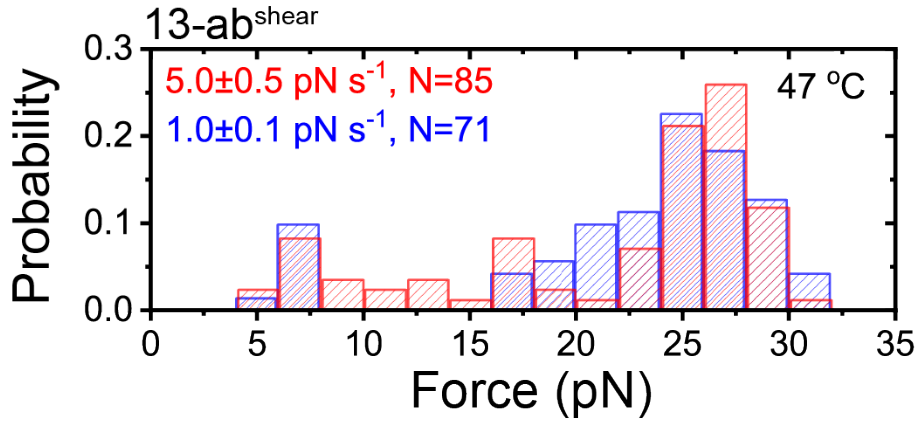

Supplementary Figure 12. Mechanical stability of 13-ab^{shear} at 47°C. Normalized histograms of the 13-ab^{shear} helix-heterotetramer rupture forces distribution at force loading rates of 1.0 ± 0.1 pN s^{-1}(blue) and 5.0 ± 0.5 pN s^{-1}(red) at 47°C. Number of data points obtained from >5 independent tethers is indicated in the figure. Source data are provided as a Source Data file.

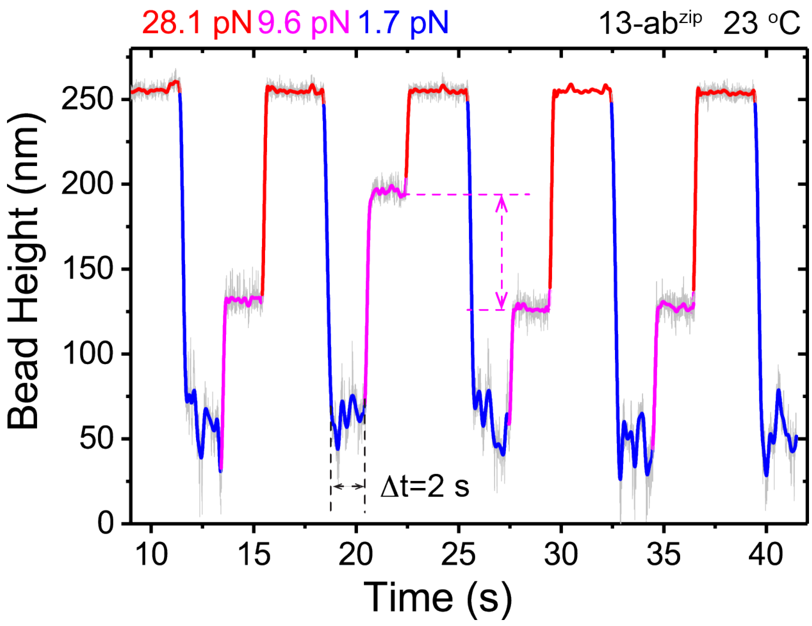

Supplementary Figure 13. Example of the force-clamping procedure to quantify the complex formation probability at low forces. Briefly, the molecule was held at a high force of ~28 pN (red color) to ensure the fully

rupture of the 13-ab^{zip} complex, and then jumped to a set of low forces (blue color, 1.7 pN in this example) and clamped at this low force for a duration of Δt (2s in this example) to allow potential formation of the complex. After the Δt force-clamping, the molecule was jumped to a force of ~9.6 pN (magenta color) for 2 second to check if the heterotetramer is formed (looped) or not during the Δt force-clamping at low forces, based on the huge extension difference between the looped and unlooped state of the molecule at this force (>50 nm, indicated by magenta dash arrow). Four example force-clamping cycles are shown in the panel, in which the molecule did not form the complex at the second cycle within the 2s clamping at low force. By repeating such procedure on multiple molecule for multiple cycles, the probability of the complex formation at each force and each low force waiting time can be obtained. The color lines show 20-FFT smooth of the raw data (grey). Source data are provided as a Source Data file.

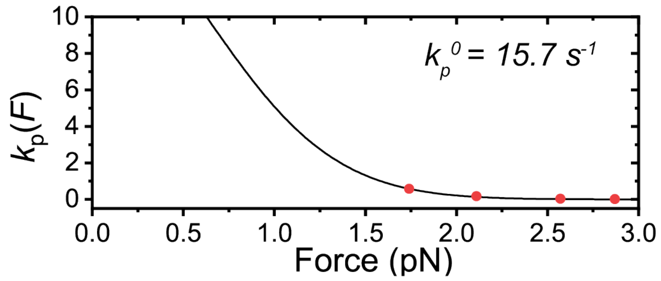

Supplementary Figure 14. Force dependent relooping rate of 13-ab^{zip} construct. The experimentally obtained data points of relooping rate at four forces are plotted as red solid cycles, which were fitted to a force-dependent expression of $k_p(F)$ based on Arrhenius Law (details in Supplementary note

7) to obtain the zero-force relooping rate k_p^0 as indicated. Source data are provided as a Source Data file.

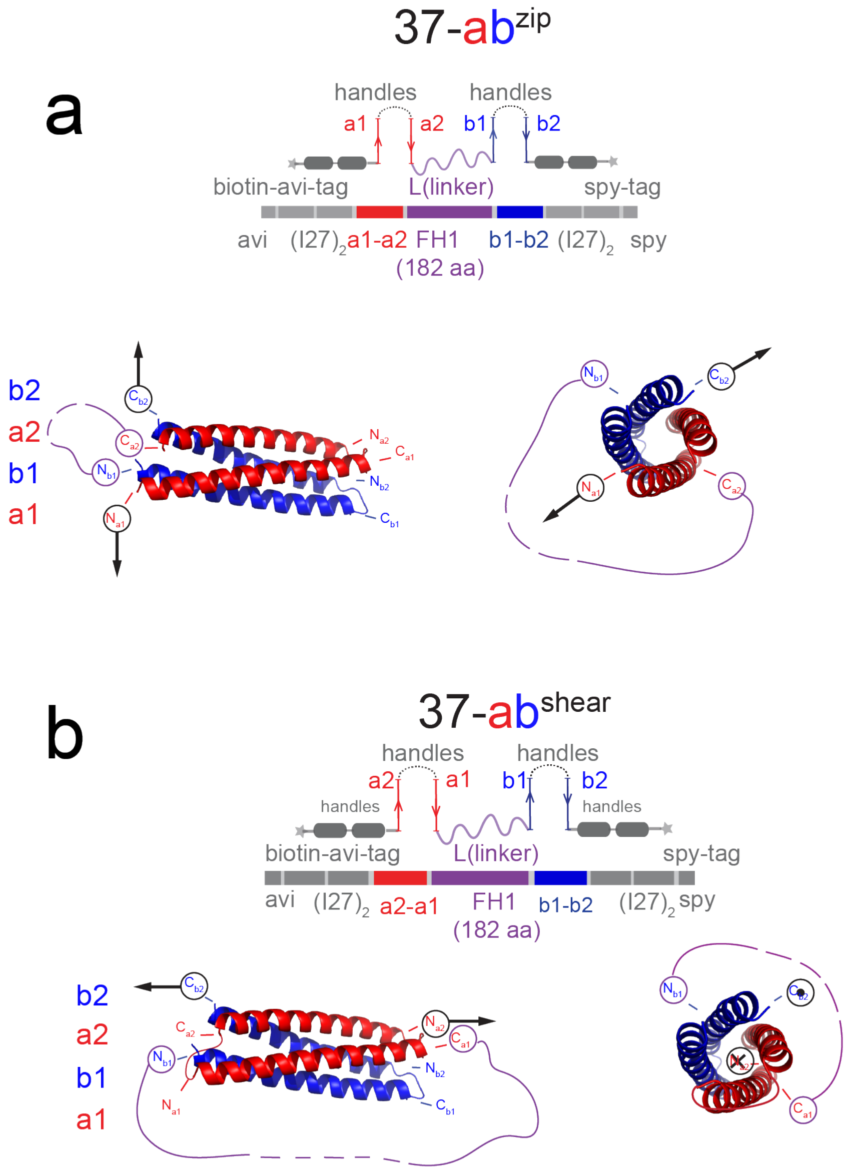

Supplementary Figure 15. The design and structure of 37-ab^{zip} and 37-ab^{shear} constructs in side and front views. (a). The structure of 37-ab^{zip} construct. Top panel: single-molecule construct of 37-ab^{zip}. Bottom panel: side

and front view of 37-ab^{zip} helix bundle. **(b).** The structure of 37-ab^{shear} construct. Top panel: single-molecule construct of 13-ab^{shear}. Bottom panel: side and front view of 37-ab^{shear} helix bundle. Black circles indicate the force-attaching points, purple circles indicate the attaching points of FH1 linker. The black arrows indicate the force direction. The black dot in the black circle indicates force pointing outward to the screen, the black cross in the black circle indicates force pointing inward into the screen.

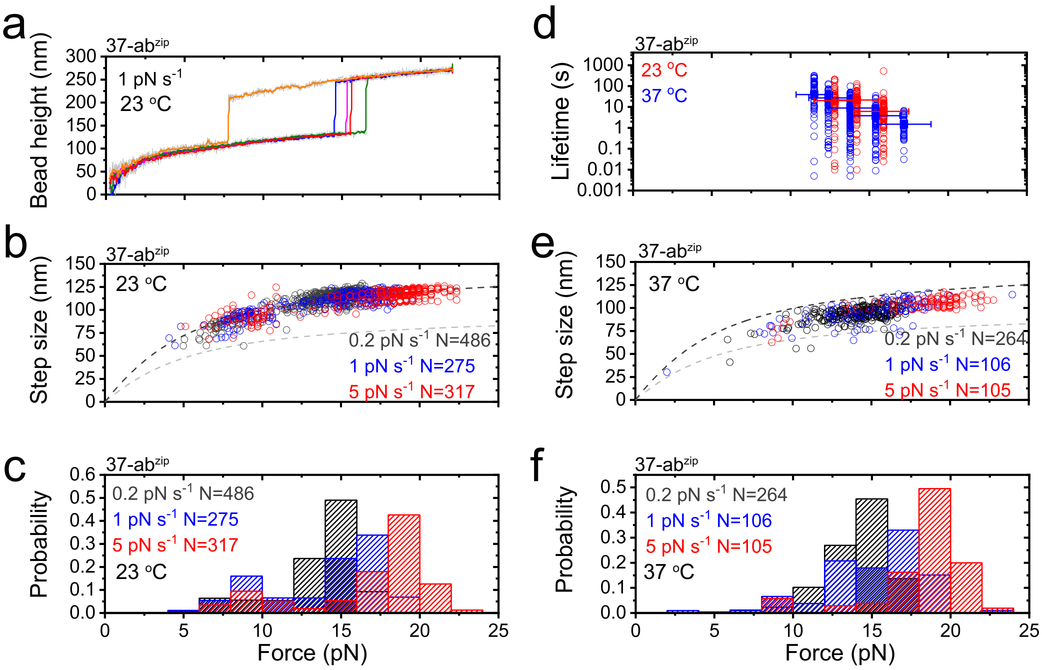

Supplementary Figure 16. Direct single-molecule quantification of the mechanical stability of #37-ab^{zip} helix-heterotetramer. (a). Five representative force-extension curves of the 37-ab^{zip} complex obtained at a force loading rate of 1 pN s^{-1} at 23 °C. The stepwise bead height jump indicates the force-induced unlooping transitions. The unlooping of 37-ab^{zip} and unfolding of a and b helix hairpins typically occur concurrently because that the unfolding

forces of the a and b helix hairpins are smaller than the unlooping forces (Supplementary Figure 17). **(b&e).** The force-step size graph of the force-dependent rupture transitions of 37-ab^{zip} complex during force-increase scans at force loading rates of 0.2 pN s^{-1} (dark grey), 1 pN s^{-1} (blue) and 5 pN s^{-1} (red) at 23 ^{o}C (**b**) or 37 ^{o}C (**e**). Number of data points obtained from >5 independent tethers is indicated in the figure panel. The dash curves are the theoretical calculations of the force-step size curves of the unlooping transition based on polymer models. The dark grey curve is calculated with an assumption that the unfolded state of ab^{zip} exists as a fully unstructured peptide chain; the light grey curve is calculated with an assumption that the unfolded state of ab^{zip} is a combination of a chain of two helix hairpins (a and b) and a fully unstructured peptide chain of FH1 region. More details of the theoretical models can be found in Supplementary Note 6, Supplementary Figures 18&19. **(c&f).** Normalized histograms of the 37-ab^{zip} complex unlooping forces distribution at force loading rates of 0.2 pN s^{-1}(dark grey), 1 pN s^{-1}(blue) and 5 pN s^{-1}(red) at 23 ^{o}C (**c**) or 37 ^{o}C (**f**). Number of data points obtained from >5 different molecules is indicated in the figure. **(d).** Force dependent lifetimes of 37-ab^{zip} complex at 23 ^{o}C (red) and 37 ^{o}C (blue). The solid squares represent the single exponential decay fitted mean lifetime of distribution histogram. The hollow circles represent each individual lifetime measured. Around 50 lifetimes were measured for each force. The horizontal error bars indicate 10% of relative force calibration uncertainty of the system. (see details in Materials and

Methods section). The vertical error bars indicate fitted standard errors. The lifetime distribution of all the force are show in Supplementary Figure 20&21. Source data are provided as a Source Data file.

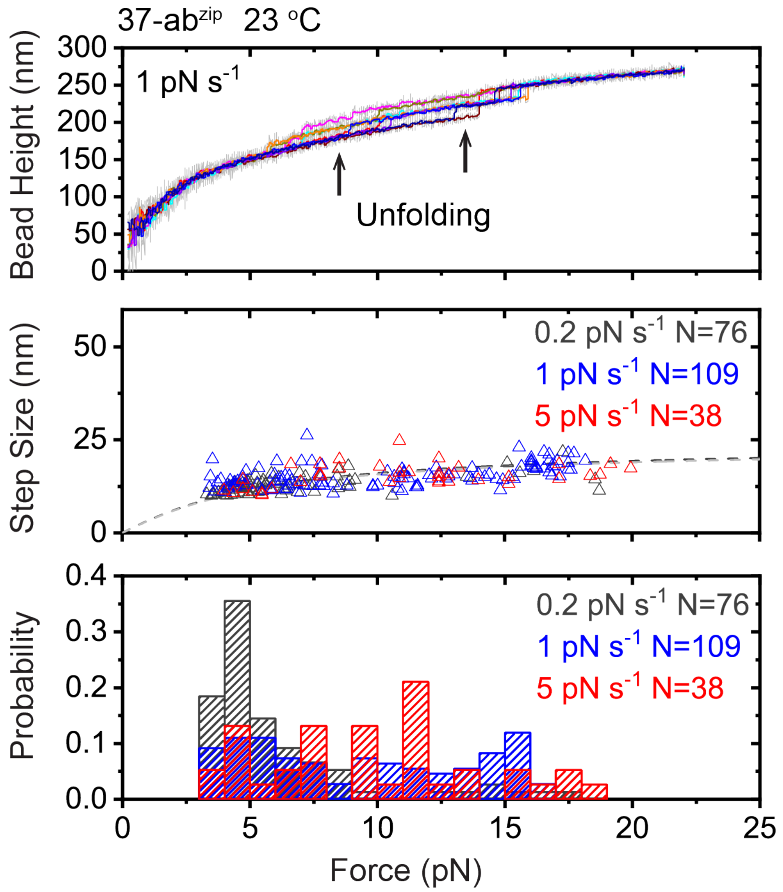

Supplementary Figure 17. Mechanical stability of a and b helix hairpins in 37-ab^{zip}. (a). Nine representative force bead height curves of the 37-ab^{zip} construct during force-increase scans started from unlooping state with a loading rate of 1 pN s^{-1}. The color lines show 20-FFT smooth of the raw data (grey). **(b).** The force-step size graph of the force-dependent unfolding of a and b helix hairpins in 37-ab^{zip} during force-increase scans with loading rates of 0.2 pN s^{-1} (dark grey), 1 pN s^{-1}(blue), and 5 pN s^{-1}(red), respectively. Number of

data points obtained from >5 different molecules is indicated in the panel. The dash curves are the theoretical calculations of the force dependent unfolding step sizes of a and b. More details of the theoretical calculation can be found in Supplementary Note 6, Supplementary Figures 16&17. **(c).** Normalized histograms of the a and b hairpins unfolding force distributions at loading rates of 0.2 pN s^{-1} (dark grey), 1 pN s^{-1}(blue), and 5 pN s^{-1}(red). Source data are provided as a Source Data file.

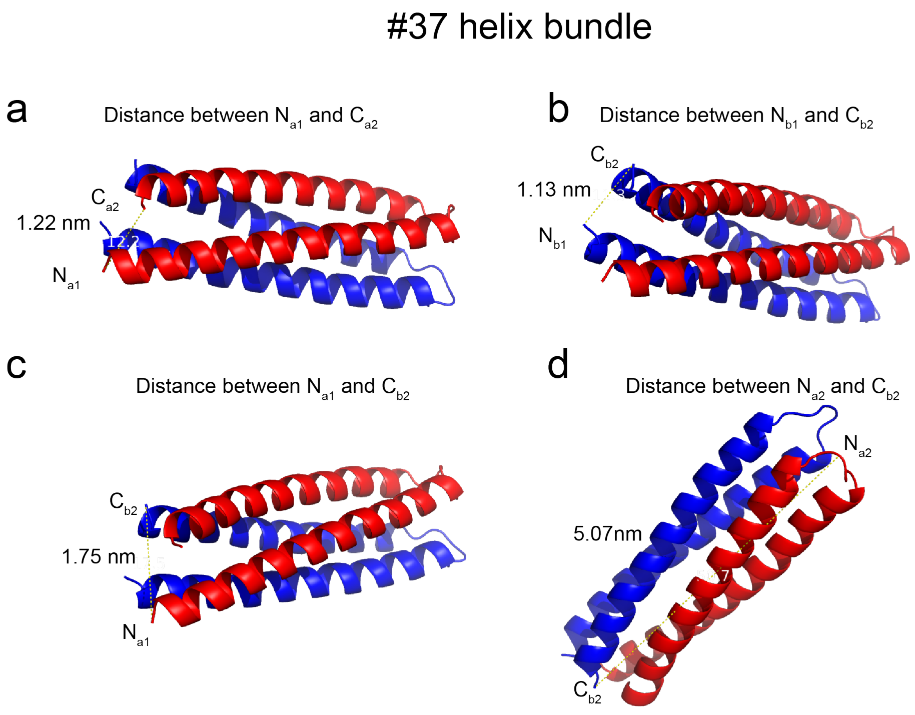

Supplementary Figure 18. The rigid body length b_0 of the folded #37 helix bundle. The b_0 **of** 37-a helix hairpin **(a)**, 37-b helix hairpin **(b)**, the folded 37-ab^{zip} complex **(c)**, and the 37-ab^{shear} complex **(d),** are measured by pymol based on the structure, and indicated in on the panels.

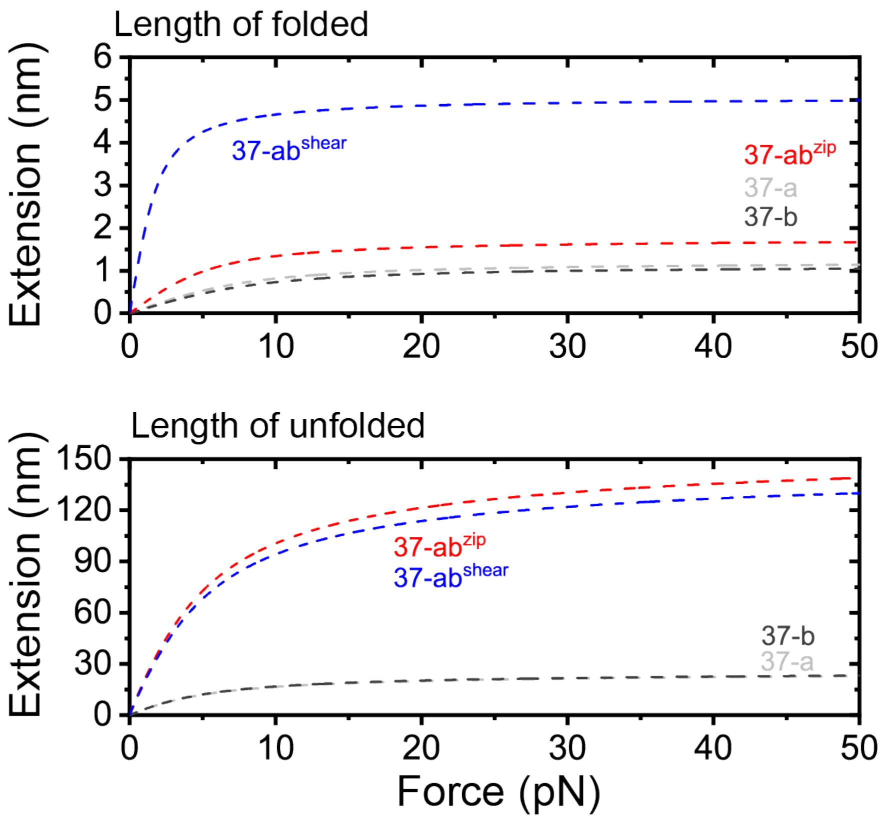

Supplementary Figure 19. Theoretical force-extension curves of the #37 helix bundle. Top panel: theoretical force-extension curve of the folded 37-a helix hairpin (grey), 37-b helix hairpin (dark grey), 37-ab^{zip} helix bundle (red), 37-ab^{shear} helix bundle (blue). Bottom panel: theoretical force-extension curve of the unfolded 37-a helix bundle (grey), 37-b helix bundle (dark grey), 37-ab^{zip} helix bundle (red), 37-ab^{shear} helix bundle (blue). Details of the theoretical models and assumptions are included in Supplemenrary Note 6. Source data are provided as a Source Data file.

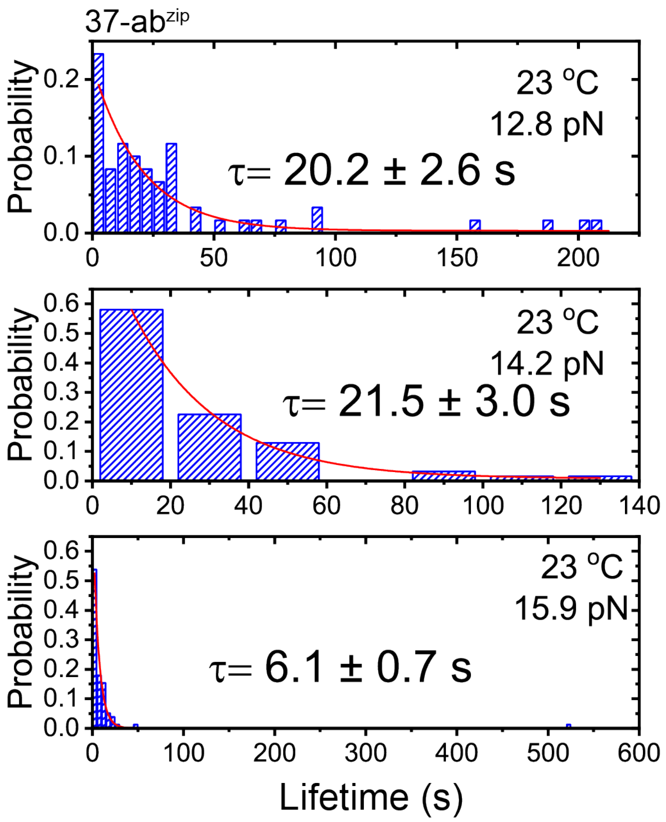

Supplementary Figure 20. Lifetime distribution of the 37-ab^{zip} at different forces, at 23 °C. The red curve is fitted curve of exponential decay function to the normalized lifetime distribution. The forces, fitted mean, and standard error are indicated in each panel. The number of data points obtained at 12.8 pN, 14.2 pN, and 15.9 pN are 62, 78, and 60, respectively. Source data are provided as a Source Data file.

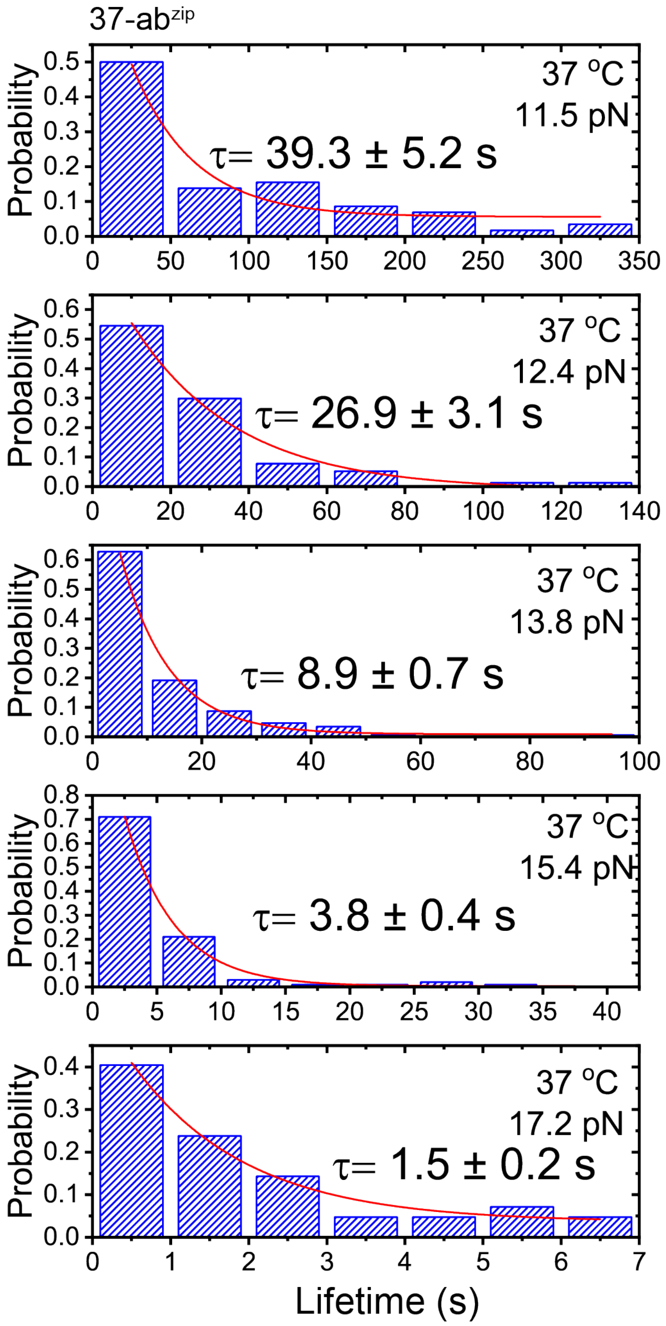

Supplementary Figure 21. Lifetime distribution of the 37-ab^{zip} at different forces, at 37 °C. The red curve is fitted curve of exponential decay function to the normalized lifetime distribution. The forces, fitted mean, and standard error are indicated in each panel. The number of data points obtained at 11.5 pN, 12.4 pN, 13.8 pN, 15.4 pN and 17.2 pN are 58, 77, 172, 100, and 42,

respectively. Source data are provided as a Source Data file.

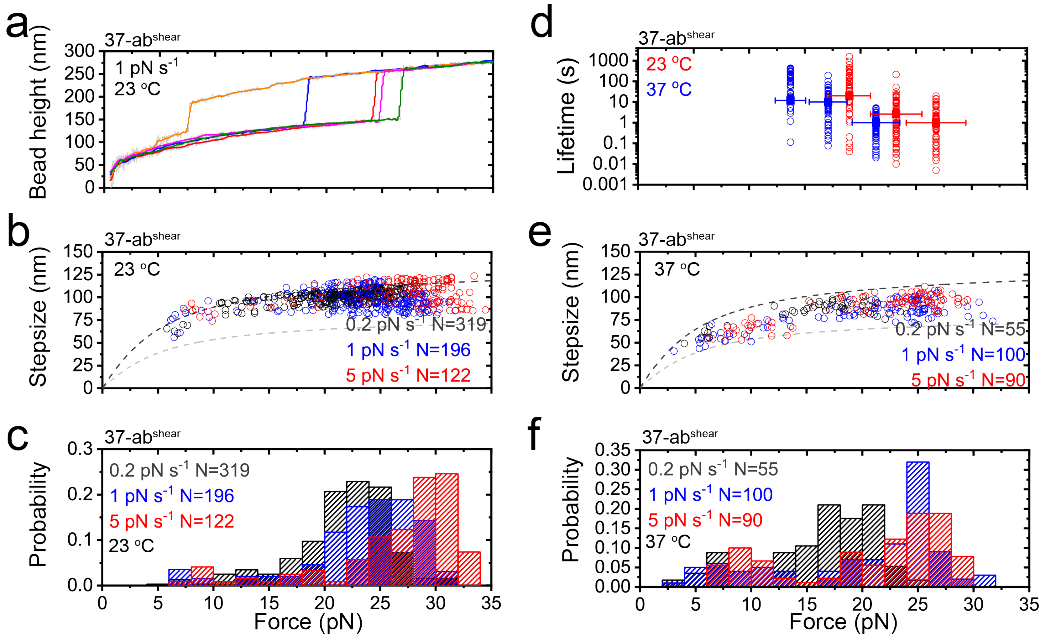

Supplementary Figure 22. Direct single-molecule quantification of the mechanical stability of #37-ab^{shear} helix-heterotetramer. (a). Five representative force bead height curves of the 37-ab^{shear} complex during force-increase scans with a force loading rate of 1 pN s^{-1} at 23 °C. The unlooping event in each trace is indicated by a big stepwise bead height jump. **(b&e).** The force-step size graph of the force-dependent rupture transitions of 37-ab^{shear} complex during force-increase scans at force loading rates of 0.2 pN s^{-1} (dark grey), 1 pN s^{-1} (blue) and 5 pN s^{-1} (red) at 23 °C (**b**) or 37 °C (**e**). Number of data points obtained from >5 different molecules is indicated in the figure panels. The dash curves are the theoretical calculations of the force-step size curves of the unlooping transition. The dark grey curve is calculated with an assumption that the unfolded state of 37-ab^{shear} is a fully unstructured peptide

polymer chain; the light grey curve is calculated with an assumption that the unfolded state of 37-ab^{shear} is a combination of a chain of two helix hairpins (a and b) and a fully unstructured peptide chain of FH1 region. More details of the theoretical calculation can be found in Supplementary Note 6, Supplementary Figures 18&19. **(c&f).** Normalized histograms of the unlooping forces of 37-ab^{shear} complex with force loading rates of 0.2 pN s^{-1}(dark grey), 1 pN s^{-1}(blue) and 5 pN s^{-1}(red) at 23 ^{o}C (**c**) or 37 ^{o}C (**f**). **(d).** Force dependent lifetimes of 37-ab^{shear} complex at 23 ^{o}C (red) and 37 ^{o}C (blue). The solid squares represent the single exponential decay fitted mean lifetime of distribution histogram. The hollow circles represent each individual lifetime measured. Around 50 lifetimes were measured for each force. The horizontal error bars indicate 10% of relative force calibration uncertainty of the system. (see details in Materials and Methods section). The vertical error bars indicate fitted standard errors. The lifetime distribution of all the force are show in Supplementary Figure 23&24. Source data are provided as a Source Data file.

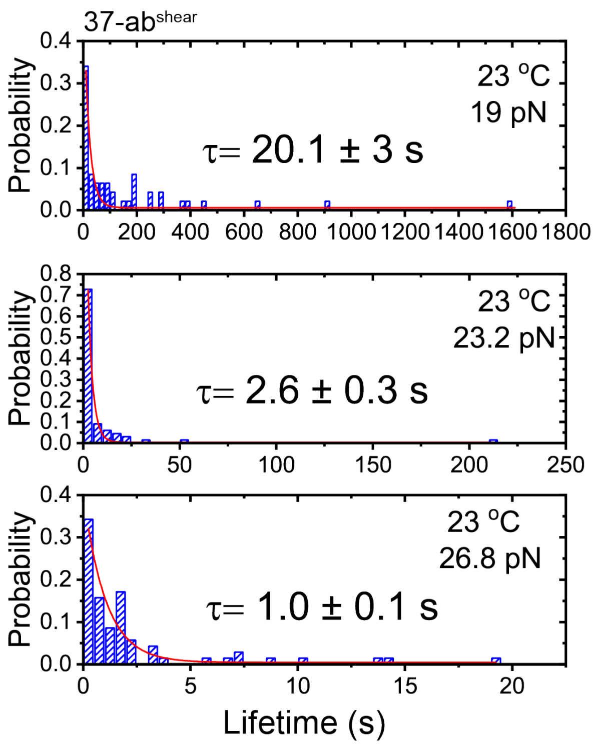

Supplementary Figure 23. Lifetime distribution of the 37-ab^{shear} at different forces, at 23 °C. The red curve is fitted curve of exponential decay function. The forces, fitted mean, and standard error are indicated in each figure. The number of data points obtained at 19 pN, 23.2 pN, and 26.8 pN are 47, 66, and 70, respectively. Source data are provided as a Source Data file.

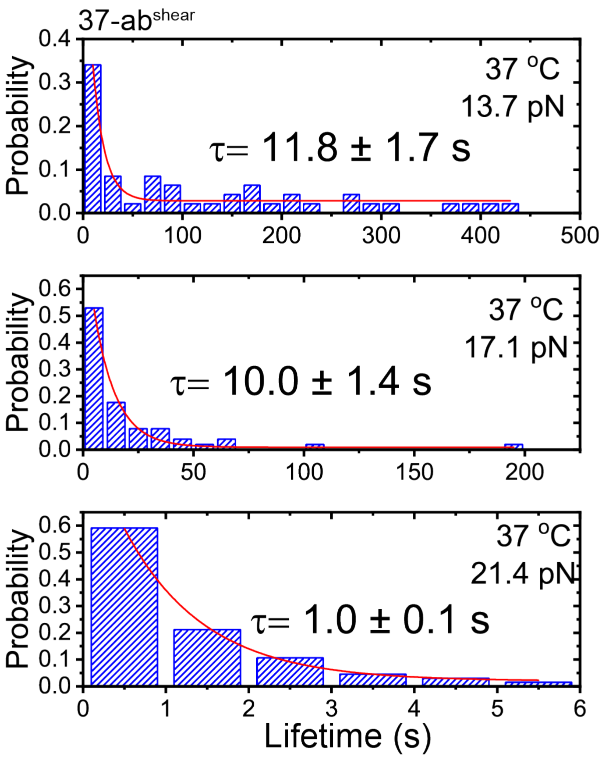

Supplementary Figure 24. Lifetime distribution of the 37-ab^{shear} at different forces, at 37 °C. The red curve is fitted curve of exponential decay function. The forces, fitted mean, and standard error are indicated in each figure. The number of data points obtained at 13.7 pN, 17.1 pN, and 21.4 pN are 47, 51, and 66, respectively. Source data are provided as a Source Data file.

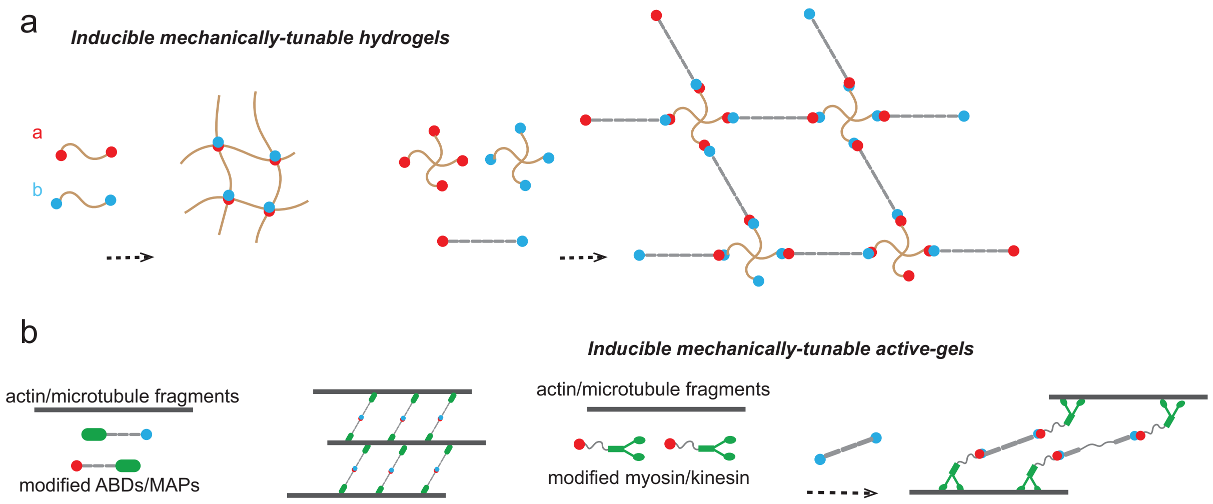

Supplementary Figure 25. The orthogonal heterodimerization system based hydrogels and active materials. (a). Mechanically-tunable hydrogels formed by heterodimerization of a and b helix-hairpins. Left panel: hydrogel is formed by a and b helix-hairpins that directly anchored to the hydrogel filaments. Right panel: hydrogel is formed by a and b helix-hairpins that linked each other through flexible linker or rigid spacer. **(b).** Inducible mechanically-tunable active-gels formed by orthogonal a and b helix-hairpins heterodimers. We note that the schematics are drawn in a highly ordered manner to highlight the design principles. The networks formed in actual hydrogels are expected to be much more disordered.

Supplementary Table

Force	$k_{\mathrm{p}}(F)$	$k_{\mathrm{u}}(F)$	k_{p}^{0}
1.74	0.5705	0.0198	15.4059
2.11	0.1826	0.0052	21.2497
2.57	0.0392	0.0072	38.5523
2.87	0.0111	0.0142	52.2072

Supplementary Table 1: Kinetic parameters of the force-dependent looping and unlooping rates of the 13-ab^{zip} construct. The table summarizes the fitted force-dependent looping and unlooping rates $k_{\mathrm{p}}(F)$ and $k_{\mathrm{u}}(F)$, the extrapolated zero-force re-pairing rates k_{p}^{0} was obtained by fitting the $k_{\mathrm{p}}(F)$ to the approximated Arrhenius Law equation as described in Supplementary Note 7.

Supplementary Notes

Supplementary Note 1. Plasmids constructs and protein expression.

Plasmids constructs. The DNA fragments encoding the sequences of helix hairpins 13-a1a2, 13-a2a1, 13-b1b2, 13-a2, 37-a1a2, 37-a2a1, 37-b1b2 [1], avi-tag (GLNDIFEAQKIEWHE), spy-tag(AHIVMVDAYKPTK) [2] and FH1 domain (residues 582-764 of DIAP1_HUMAN) [3] were synthesized by GeneArt (Thermo Fisher) or gBlock (IDT). The corresponding DNA fragments were then sub-cloned into expression vector pET151-avi-2I27-A-FH1-B-2I27-spy [4-6] using HiFi DNA Assembly (NEB), where A is to be replaced with the 13-a1a2, 13-a2a1, or 13-a2, B with the 13-b1b2 to make single molecule constructs: 13-ab^{zip}, 13-ab^{shear}, or 13-a^{half}b, respectively. Similarly, A is to be replaced with the 37-a1a2 or 37-a2a1, and B with the 37-b1b2 to make single molecule constructs: 37-ab^{zip} or 37-ab^{shear}, respectively.

The DNA fragments encoding four repeats of titin I27 domain (4xI27) and six repeats of the spectrin-repeat domains (6xSR) were previously synthesized by geneArt [7]. The DNA fragments were then assembled with 13-a2a1 into the pET151-avi vector by HiFi DNA Assembly (NEB), resulting in avi-4xI27-a2a1, and avi-6xSR-a2a1, respectively. The DNA fragment of 13-b1b2 and spy-tag were assembled into the pET151 vector, resulting in a2a1-spy construct by HiFi DNA Assembly. All resulting plasmids were confirmed by sequencing (by 1st BASE).

Protein expression. Each plasmid that contains avi-tag was co-transformed with a BirA plasmid and expressed in Escherichia coli BL21 (DE3) cultured in LB-media with D-Biotin (Sigma Aldrich), and affinity purified through 6His-tag. The plasmids that do not contain avi-tag were transformed and expressed in Escherichia coli BL21 (DE3) cultured in LB-media, and affinity purified through 6His-tag.

Detailed sequences information of the constructs. Detailed sequences of the single-molecule constructs 13-ab^{zip}, 13-ab^{shear}, 13-a^{half}b, 37-ab^{zip}, and 37-ab^{shear} are listed below with different colors. In addition, there are short flexible linkers between two neighboring components to allow flexibility of the domains.

1. 13-ab^{zip}: avi-I27-I27-a1a2-FH1-b1b2-I27-I27-spy:

GLNDIFEAQKIEWHEGGGSGLIEVEKPLYGVEVFVGETAHFEIELSEPDVHG

QWKLKGQPLAASPDAEIIEDGKKHILILHNAQLGMTGEVSFQAANTKSAANL

KVKELGGGSGLIEVEKPLYGVEVFVGETAHFEIELSEPDVHGQWKLKGQPLA

ASPDAEIIEDGKKHILILHNAQLGMTGEVSFQAANTKSAANLKVKELGGGSG

KLGGGSGGTKEDILERQRKIIERAQEIHRRQQEILEELERIIRKPGSSEEAMK

RMLKLLEESLRLLKELLELSEESAQLLYEQRGGGSGEFPPAPPLPGDSGTIIP

PPPAPGDSTTPPPPPPPPPPPPLPGGVCISSPPSLPGGTAISPPPPLSGDA

TIPPPPPLPEGVGIPSPSSLPGGTAIPPPPPLPGSARIPPPPPPLPGSAGIPPP

PPPLPGEAGMPPPPPPLPGGPGIPPPPPFPGGPGIPPPPPGMGMPPPPPF

GFGVPAAPVLPGSGGGSGGTEKRLLEEAERAHREQKEIIKKAQELHRRLEEI

VRQSGSSEEAKKEAKKILEEIRELSKRSLELLREILYLSQEQKGSLVPRGGGS

GLEGGGSGLIEVEKPLYGVEVFVGETAHFEIELSEPDVHGQWKLKGQPLAAS

PDAEIIEDGKKHILILHNAQLGMTGEVSFQAANTKSAANLKVKELGGGSGLIE

VEKPLYGVEVFVGETAHFEIELSEPDVHGQWKLKGQPLAASPDAEIIEDGKK

HILILHNAQLGMTGEVSFQAANTKSAANLKVKELGGGSGAHIVMVDAYKPTK

2. 13-ab^{shear}: avi-I27-I27- a2---a1-FH1-b1b2-I27-I27-spy:

GLNDIFEAQKIEWHEGGGSGLIEVEKPLYGVEVFVGETAHFEIELSEPDVHG

QWKLKGQPLAASPDAEIIEDGKKHILILHNAQLGMTGEVSFQAANTKSAANL

KVKELGGGSGLIEVEKPLYGVEVFVGETAHFEIELSEPDVHGQWKLKGQPLA

ASPDAEIIEDGKKHILILHNAQLGMTGEVSFQAANTKSAANLKVKELGGGSG

KLGGGSGSSEEAMKRMLKLLEESLRLLKELLELSEESAQLLYEQRGGGSGL

EKVKKPAVPEPPPPKPVEEVEVPTVTKRERKIPEPTKVPEIKPAIPLPAPEPKP

KGGGSGGTKEDILERQRKIIERAQEIHRRQQEILEELERIIRKPGGSGGEFPP

APPLPGDSGTIIPPPPAPGDSTTPPPPPPPPPPPPPLPGGVCISSPPSLPGGT

AISPPPPLSGDATIPPPPPLPEGVGIPSPSSLPGGTAIPPPPPLPGSARIPPPP

PPLPGSAGIPPPPPPPLPGEAGMPPPPPPLPGGPGIPPPPPFPGGPGIPPPPP

GMGMPPPPPFGFGVPAAPVLPGGSGGTEKRLLEEAERAHREQKEIIKKAQE

LHRRLEEIVRQSGSSEEAKKEAKKILEEIRELSKRSLELLREILYLSQEQKGSL

VPRGGGSGLEGGGSGLIEVEKPLYGVEVFVGETAHFEIELSEPDVHGQWKL

KGQPLAASPDAEIIEDGKKHILILHNAQLGMTGEVSFQAANTKSAANLKVKEL

GGGSGLIEVEKPLYGVEVFVGETAHFEIELSEPDVHGQWKLKGQPLAASPD

AEIIEDGKKHILILHNAQLGMTGEVSFQAANTKSAANLKVKELGGGSGAHIVM

VDAYKPTK

3. 13-a^{half}b: avi-I27-I27- a2-FH1-b1b2-I27-I27-spy

GLNDIFEAQKIEWHEGGGSGLIEVEKPLYGVEVFVGETAHFEIELSEPDVHG

QWKLKGQPLAASPDAEIIEDGKKHILILHNAQLGMTGEVSFQAANTKSAANL

KVKELGGGSGLIEVEKPLYGVEVFVGETAHFEIELSEPDVHGQWKLKGQPLA

ASPDAEIIEDGKKHILILHNAQLGMTGEVSFQAANTKSAANLKVKELGGGSG

KLGSSEEAMKRMLKLLEESLRLLKELLELSEESAQLLYEQRGGGSGEFPPAP

PLPGDSGTIIPPPPAPGDSTTPPPPPPPPPPPPPLPGGVCISSPPSLPGGTAI

SPPPPLSGDATIPPPPPLPEGVGIPSPSSLPGGTAIPPPPPLPGSARIPPPPPP

LPGSAGIPPPPPPLPGEAGMPPPPPPLPGGPGIPPPPPFPGGPGIPPPPPG

MGMPPPPPFGFGVPAAPVLPGSGGGSGGTEKRLLEEAERAHREQKEIIKKA

QELHRRLEEIVRQSGSSEEAKKEAKKILEEIRELSKRSLELLREILYLSQEQKG

SLVPRGGGSGLEGGGSGLIEVEKPLYGVEVFVGETAHFEIELSEPDVHGQW

KLKGQPLAASPDAEIIEDGKKHILILHNAQLGMTGEVSFQAANTKSAANLKVK

ELGGGSGLIEVEKPLYGVEVFVGETAHFEIELSEPDVHGQWKLKGQPLAASP

DAEIIEDGKKHILILHNAQLGMTGEVSFQAANTKSAANLKVKELGGGSGAHIV

MVDAYKPTK

4. 37-ab^{zip}: avi-I27-I27- a1a2-FH1-b1b2-I27-I27-spy:

GLNDIFEAQKIEWHEGGGSGLIEVEKPLYGVEVFVGETAHFEIELSEPDVHG

QWKLKGQPLAASPDAEIIEDGKKHILILHNAQLGMTGEVSFQAANTKSAANL

KVKELGGGSGLIEVEKPLYGVEVFVGETAHFEIELSEPDVHGQWKLKGQPLA

ASPDAEIIEDGKKHILILHNAQLGMTGEVSFQAANTKSAANLKVKELGGGSG

KLGSGDSDEHLKKLKTFLENLRRHLDRLDKHIKQLRDILSENPEDERVKDVID

LSERSVRIVKTVIKIFEDSVRKKEGSGSGGSGSGGGGSLVPRGRERKIPEPT

KVPEIKPAIPLPAPEPKPKSGGGGSGGGSEGGGEFPPAPPLPGDSGTIIPPP

PAPGDSTTPPPPPPPPPPPPPLPGGVCISSPPSLPGGTAISPPPPLSGDATIP

PPPPLPEGVGIPSPSSLPGGTAIPPPPPLPGSARIPPPPPPLPGSAGIPPPPP

PLPGEAGMPPPPPPLPGGPGIPPPPPFPGGPGIPPPPPGMGMPPPPPFGF

GVPAAPVLPGSLEKVKKPAVPEPPPPKPVEEVEVPTVTKGGGSEGGSGSGG

SGSGGSDDKELDKLLDTLEKILQTATKIIDDANKLLEKLRRSERKDPKVVETY

VELLKRHEKAVKELLEIAKTHAKKVELEGGGSGLIEVEKPLYGVEVFVGETAH

FEIELSEPDVHGQWKLKGQPLAASPDAEIIEDGKKHILILHNAQLGMTGEVSF

QAANTKSAANLKVKELGGGSGLIEVEKPLYGVEVFVGETAHFEIELSEPDVH

GQWKLKGQPLAASPDAEIIEDGKKHILILHNAQLGMTGEVSFQAANTKSAAN

LKVKELGGGSGAHIVMVDAYKPTK

5. 37-ab^{shear}: avi-I27-I27- a2-a1-FH1-b1b2-I27-I27-spy:

GLNDIFEAQKIEWHEGGGSGLIEVEKPLYGVEVFVGETAHFEIELSEPDVHG

QWKLKGQPLAASPDAEIIEDGKKHILILHNAQLGMTGEVSFQAANTKSAANL

KVKELGGGSGLIEVEKPLYGVEVFVGETAHFEIELSEPDVHGQWKLKGQPLA

ASPDAEIIEDGKKHILILHNAQLGMTGEVSFQAANTKSAANLKVKELGGGSG

KLGSGEDERVKDVIDLSERSVRIVKTVIKIFEDSVRKKEGSGSGGSGSGGGG

DSDEHLKKLKTFLENLRRHLDRLDKHIKQLRDILSENPSLVPRGSGGGGSGG GSEGGGEFPPAPPLPGDSGTIIPPPPAPGDSTTPPPPPPPPPPPPPPLPGGVC ISSPPSLPGGTAISPPPPLSGDATIPPPPPLPEGVGIPSPSSLPGGTAIPPPPP LPGSARIPPPPPPLPGSAGIPPPPPPLPGEAGMPPPPPPLPGGPGIPPPPPF PGGPGIPPPPPGMGMPPPPPFGFGVPAAPVLPGSLEKVKKPAVPEPPPPKP VEEVEVPTVTKGGGSEGGSGSGGSGSGGSDDKELDKLLDTLEKILQTATKII DDANKLLEKLRRSERKDPKVVETYVELLKRHEKAVKELLEIAKTHAKKVELEG GGSGLIEVEKPLYGVEVFVGETAHFEIELSEPDVHGQWKLKGQPLAASPDAE IIEDGKKHILILHNAQLGMTGEVSFQAANTKSAANLKVKELGGGSGLIEVEKP LYGVEVFVGETAHFEIELSEPDVHGQWKLKGQPLAASPDAEIIEDGKKHILILH NAQLGMTGEVSFQAANTKSAANLKVKELGGGSGAHIVMVDAYKPT

Supplementary Note 2. Single molecule manipulation by magnetic-tweezer

Experimental setup and solution conditions. All in vitro single molecule stretching experiments were performed using a vertical magnetic-tweezer setup [8]. The channel is combined with a disturbance-free, rapid solution-exchange method to avoid flow-drag during flow exchange [9]. Experiments were performed in standard solution containing: 1X PBS, 1% BSA, 2 mM DTT, 10 mM sodium L-ascorbate at multiple temperatures (23 °C, 27 °C, 29 °C, 31 °C and 37 °C). The temperature was controlled by an objective heating system

(Bioptechs).

Surface-modification for spycatcher coating. The bottom coverslip surface of the channel is modified for specific molecule tethering. The coverslip surface was first treated with 3-Triethoxysilylpropylamine (APTES, 1% in Methanol) for 1 hour and then washed and dried by 150 ^{o}C in oven. The sample channels prepared with the APTES-coated bottom coverslip were then 1). treated with glutaraldehyde (1% in 1XPBS) for 2 hours and then washed at 23 ^{o}C; 2). incubated with spycatcher (0.05 mg/ml in 1XPBS) for 24 hours at 23 ^{o}C; 3). then incubated with BSA (3% BSA in 1XPBS) for 24 hours at 23 ^{o}C for surface blocking. The BSA-blocked spycatcher-coated channels were then stored at 4 ^{o}C for use for up to two months.

Specific target molecule tethering. To form the specific tethers of the target molecule (such as 13-ab^{zip}, 13-ab^{shear}, 13-a^{half}b, 37-ab^{zip}, or 37-ab^{shear}) in sample channel, the target molecule was first diluted to ~ 10^{-4} mg/ml in the standard solution and flowed into the sample channel (coated with spycatcher) to allow the specific spytag-spycatcher interaction during ~20 min incubation. The un-tethered molecules were washed away, followed by 5 min incubation of ~ 10^{-2} mg/ml neutravidin in standard solution, during which the neutravidin binds to the biotin on the N-terminus of the target molecule. The un-bound neutravidins were then washed away, followed by ~ 20 min incubation of biotin-572-bp-dsDNA linked super-paramagnetic beads (2.8 μm in diameter, ~ 0.1

mg/ml in standard solution). During the incubation, the biotin on the DNA can binds to the remaining binding sites on neutravidin on the target molecule. After the incubation, the unbound beads were gently washed way. Here we note that, the 572-bp DNA handle was used as a spacer to increase the distance between the bead surface and bottom coverslip surface to avoid non-specific interaction between the two surfaces. The DNA handle also serves as an additional specific control since the DNA handle has a signature signal at ~65 pN, termed as force-induced overstretching transition, where the extension sudden increases to 1.6-1.7 times of the contour length of the B-form DNA [10].

In the demonstration experiments of the mechanics of I27 and spectrin-repeat domains, the b1b2-spy molecule (~ 0.1 mg/ml) in standard solution was first incubated with the spycatcher-coated sample channel for ~ 20 min to coat the b1b2 on the bottom surface (the unbound molecule was then washed away). Then the avi-4xI27-a2a1 or avi-6xSR-a2a1 molecule (~ 10^{-4} mg/ml in standard solution) was incubated in the channel to allow specific a-b helix-bundle interaction between the split-13-ab^{shear}. Then similar neutravidin incubation and beads incubation steps (detailed in the above paragraph) were performed. Here we note that these experiments were mainly carried out to demonstrate that the specific tethers formed between the split helix-pairs can be used for single-molecule protein mechanics studies in various temperatures. Variations of the a-b tethering methods can be utilized for other applications.

Bead-height determination of the magnetic-tweezer setup. In the magnetic tweezer experiments, a single target protein was tethered between a 2.8-μm-diameter superparamagnetic bead and the bottom coverslip surface. What we recorded was the height of the bead from the coverslip surface along the force direction [8]. During a force change, the height change of the bead includes contributions from both extension change of the molecule and the bead re-orientation due to torque rebalance after the force change. Hence, a force jump (which typically took ≤0.25 s in our setup) was typically accompanied with a stepwise bead height change, which magnitude depends on the level of bead rotation due to torque rebalance and the extension change of the molecule. On the other hand, at a fixed force, the torque remains balanced. Therefore, the bead height change at a fixed force equals the extension change of the molecule. In addition, during force-increase/decrease scans at loading rates of 0.2 pN/s to 5 pN/s, the force change during the stepwise bead height change (which occurs within a time window of <0.01 s, i.e., the temporal resolution of our setup) is negligible (≤0.002 to 0.05 pN). Hence, the force-dependent stepwise bead height change during linear force scans also equals the extension change resulted from structural changes of the molecule.

Supplementary Note 3. Negligible mechanical perturbation exerted by looped flexible peptide chain linker to the complex

The total number of residues of the linker is ~ 200 a.a., including the 182 a.a. of FH1 and two additional short flexible linkers on two sides of the FH1. Detailed sequence information is provided in the Supplementary Note 1. The bending persistence of the linker is only ~0.8 nm [3].

For ab^{zip} complex, the FH1 linker links the C-terminus of a2 helix and N-terminus of b1 helix. The end-to-end distance of the looped long flexible linker is ~1 nm estimated based on the structure. Based on the force-extension curve of the linker using worm-like-chain model [11] with a bending persistence length of ~0.8 nm, the looped linker exerts a force of < 0.1 pN, which is about 100-fold smaller than the rupture forces of the ab^{zip} complex in our experiments.

For the ab^{shear} complex, the FH1 linker links the C-terminus of a1 helix and N-terminus of b1 helix. The end-to-end distance of the looped long flexible linker is ~5 nm estimated based on the structure. Based on the force-extension curve of the linker using worm-like-chain model [11], the looped linker exerts a force of < 0.6 pN to the complex, which is about 20-fold smaller than the rupture forces of the ab^{shear} observed in our experiments. Similarly, the FH1 linker links the C-terminus of a2 helix and N-terminus of b1 helix in the $a^{half}b$, the end-to-end distance of the looped long flexible linker is ~1 nm estimated based on the structure. Based on the force-extension curve of the linker using worm-like-chain model [11], the looped linker exerts a force of < 0.1 pN, which is about 100-fold smaller than the rupture forces of the $a^{half}b$ complex in our experiments.

Together, these calculations suggest that due to the highly flexible nature of the long unstructured peptide chain, the long linker does not introduce significant mechanical perturbation to the complex. Hence, it does not affect the quantification of the mechanical stability of the helix-bundles in our study.

Supplementary Note 4. Differential force-responses of I27 domain and the helix-heterotetramer

The four repeats of I27 domain in the ab^{zip}, ab^{shear} and $a^{half}b$ were used as molecular spacers and specificity control. The force responses of I27 at room temperature has been well characterized [12], it has a very slow unfolding rate (~10^{-3} s^{-1}) within forces <50 pN associated with a force-dependent unfolding step size of ~10-24 nm [12]. Such signature unfolding signal does not affect the determination of the complex rupture signal.

The mechanical rupturing of the helix-bundles exhibits a slip-bond kinetics (i.e., the lifetime decreases as force increases), which involves a rather large transition distance in 1-5 nm (e.g., ~4.5 nm for ab^{zip}, ~1.8 nm for ab^{shear}, and ~1.3 nm for $a^{half}b$). Therefore, the bundle lifetime decreases as force increases. At forces > 30 pN, the lifetime decreases to ~ 10 seconds.

In contrast, the titin I27 domain exhibit a catch-bond kinetics (i.e., the lifetime increases as force increases) at forces below 20 pN, which switches to a slip-bond kinetics at forces > 20 pN [12]. In the slip-bond force regime, the transition

distance of I27 is ~ 0.6 nm [12], which is much smaller than that of helix-bundle rupturing. Therefore, in the regime, the I27 lifetime decreases with a slower rate than that of the helix-bundles.

Therefore, due to the differential force-dependent lifetimes between I27 and helix-bundles, the I27 unfolding rarely occurs during typical force loading assays (e.g. Supplementary Figure 3). It also explains why I27 unfolding events were observed during long time measurement at constant forces of a few pN (Supplementary Figure 3).

Supplementary Note 5. Mechanical stability of the a and b helix hairpins

To probe the mechanical stability of the a and b helix hairpins, we performed force-increase scans on 13-ab^{zip} construct (Supplementary Figure 2). Since at low forces, the 13-ab^{zip} might exist in a looped state where the a and b form a helix-heterotetramer with the long linker looped inside, or an unlooped state where the a and b were folded into helix-hairpin structure and separated with each other. The looped state can be easily distinguished with the unlooped state by the signature signal of releasing the long linker, as shown in Figure 1 in main text. Unfolding of the a and b helix hairpins were observed for those scans started from an unlooped state, indicated by two small stepwise force-dependent unfolding steps in 10 – 20 nm, with transitions forces distributed around two peaks of ~ 6 pN and ~9 pN with a loading rate of 1 pN s^{-1}

(Supplementary Figure 2). In contrast, the unlooping forces (rupture forces of the helix-heterotetramer) distributed at ~ 12 pN, with a much larger step size (~ 80 nm), as shown in Figure 1 and Supplementary Figure 2. As the average unlooping force is greater than those of the a and b helix hairpins unfolding, the unlooping transition is always concurrent with the unfolding of the a and b helix hairpins.

After the unlooping of the 13-ab^{zip} construct, we performed force-decrease scans, and expected to observe the refolding of both the a and b helix hairpins before the relooping of the construct could take place. Indeed, as shown in the four representative time traces of the bead height in Supplementary Figure 2, refolding of the a and b helix hairpins were observed in these time traces, associated with a small stepwise height decrease in 10 – 15 nm depending on the force when the refolding occurred. In two of the time traces, relooping occurred after refolding of the a and b helix hairpins during the force-decrease scans indicated by larger step sizes at low forces. Here we note that, the unlooping or looping state of 13-ab^{zip} can be turned by value of low forces and waiting time (Figure 4).

Next, to distinguish the unfolding signals of a and b, we performed similar force-increase scans on the 13-a^{half}b construct, which contains two interacting components: the b helix hairpin and the a2 peptide with a tendency to form a helix. In the unlooped state of 13-a^{half}b, unfolding of the b helix hairpin was

observed indicated by a small force-dependent stepwise height increase in 10 – 20 nm (Supplementary Figure 2), distributed around a single peak of ~ 6 pN. Compared to the unfolding signals of 13-ab^{zip}, these results suggest that the a helix hairpin unfolds at ~ 9 pN while the b unfolds at ~ 6 pN with a loading rate of 1 pN s^{-1}.

Supplementary Note 6. **Theoretical models of force responses of the folded/unfolded protein domain/complex**

The force-extension curves of looped ab^{zip}, ab^{shear}, a^{half}b complex, and the folded titin I27 domain and spectrin-repeat domain (SR) are determined by the rigid rotation fluctuation of a characteristic rigid-body with a length b, which is the distance between the two force-attaching points (*i.e.*, the N- to C- terminal distance in our experiment). The value of b for 13-ab^{zip}, 13-ab^{shear}, 13-a^{half}b, 37-ab^{zip}, 37-ab^{shear}, I27 and SR are estimated to be 1.2 nm, 5.1 nm, 5.1 nm,1.8 nm, 5.1 nm, 4.3 nm and 6.4 nm, respectively, based on the corresponding folded structures (Supplementary Figures 18&19). These force-extension curves of these folded structures can be described by the freely-jointed chain (FJC) polymer model with a single segment: $x^{\mathrm{folded}}\,(F) = b(\coth\left(\frac{Fb}{k_BT}\right) - \frac{k_BT}{Fb})$. The resulting theoretical force extension curves of the folded helix hairpins (13-a1a2, 13-b1b2, 37-a1a2, 37-b1b2), the folded helix-heterotetramers (13-ab^{zip}, 13-ab^{shear}, 37-ab^{zip} and 37-ab^{shear}), and the demonstrating protein domains I27

and spectrin-repeat (SR) are plotted in top panels of Supplementary Figure 20 and Supplementary Figure 21.

Assuming the unfolded/unlooped states of the domains are fully unstructured flexible peptide chain, the force-extension curves of these unlooped ab^{zip}, ab^{shear}, $a^{half}b$ complex, and unfolded I27 and SR domains can be described by the worm-like chain (WLC) polymer model through the Marko–Siggia formula [11]: $\frac{FA}{k_BT} = \frac{1}{4(1-\frac{x^{\mathrm{WLC}}(F)}{l})^2} - \frac{1}{4} + \frac{x^{\mathrm{WLC}}(F)}{l}$, where $A \sim 0.8$ nm [13] is the bending persistence length of the peptide chain (A is temperature dependent, and decreases by ~5% when temperature is increased from 23°C to 37°C), $l = n * l_0$ is the contour length of the unlooped linker or the unfolded domain, n is the number of residues of the corresponding construct in the unstructured state, $l_0 = 0.38$ nm is the contour length of per residue. The resulting theoretical force extension curves of the unfolded helix hairpins (13-a1a2, 13-b1b2, 37-a1a2, 37-b1b2), the unfolded helix-heterotetramers (13-ab^{zip}, 13-ab^{shear}, 37-ab^{zip} and 37-ab^{shear}) and the unfolded demonstrating protein domains I27 and SR, are plotted in the bottom panels of Supplementary Figure 20 and Supplementary Figure 21. The transition step size of the force-dependent unfolding/unlooping event is the extension differences before and after unfolding/unlooping at the transition force, *i.e.*, $\Delta x(F) = x^{\mathrm{unfolded}}(F) - x^{\mathrm{folded}}(F)$.

Supplementary Note 7. Arrhenius Law based theoretical expression of force-dependent transition rates

The force-dependent unlooping/relooping rate of the a-b complex can be described with a general Arrhenius Law as $k^{\mathrm{Arrh}}(F) = k^{0}_{\mathrm{Arrh}} \exp[-\beta \Delta\phi^{*}_{\mathrm{Arrh}}(F)]$, where $\Delta\phi^{*}_{\mathrm{Arrh}}(F) = -\int_0^F \Delta^{*}_{\mathrm{Arrh}}(f)df$, is the free energy difference between the transition state and the initial state, $\Delta^{*}_{\mathrm{Arrh}}(F)$ can be calculated based on the force-dependent extension difference between the transition state structure and the initial state structure [4,14].

For the force-dependent un-looping transition, assuming the transition state is a partially folded structure consisting of a folded a-b helix bundle core and a small unpeeled fraction that is modeled as peptide chain involved with a number of residues, n^{*}_{Arrh}. Therefore, the transition distance for the unlooping is $\Delta^{*,\mathrm{u}}_{\mathrm{Arrh}}(F) = x^{*,\mathrm{peptide}}(F) + x^{*,\mathrm{bundle}}(F) - x^{\mathrm{folded}}(F)$, where $x^{*,\mathrm{bundle}}(F)$ and $x^{*,\mathrm{peptide}}(F)$ are the force-dependent extensions of the folded core and the peptide chain of the transition state, respectively, and $x^{\mathrm{folded}}(F)$ is the force-dependent extension of the looped complex. Here we note, if the unlooping transition occurs at sufficiently high forces, the $\Delta^{*,\mathrm{u}}_{\mathrm{Arrh}}(F)$ can be approximated to be a constant value Δ. The force-dependent unlooping rate then can be approximated to be Bell's model [15]: $k^{\mathrm{Bell}}(F) = k^{0} \exp[\beta F \Delta]$. Based on the Bell model, we estimated the Δ for 13-ab^{zip} 13-ab^{shear} and 13-a^{half}b to be 4.5±0.3 nm, 1.8±0.1 nm, and is 1.3±0.1 nm, respectively.

For the force-dependent relooping transition of the linked a-b complex, the transition distance is $\Delta_{\mathrm{Arrh}}^{*,\mathrm{p}}(F) = x^{*,\mathrm{peptide}}(F) + x^{*,\mathrm{bundle}}(F) - x^{\mathrm{unfolded}}(F)$, where $x^{\mathrm{unfolded}}(F)$ is the force-dependent extension of the unlooped complex. Since the $x^{\mathrm{unfolded}}(F)$ includes a long flexible linker. Over the force range tested in our experiments (1.7 pN-2.9 pN), $\Delta_{\mathrm{Arrh}}^{*,\mathrm{u}}(F)$ can be approximated to be a small constant Δ^{u}, while the $\Delta_{\mathrm{Arrh}}^{*,\mathrm{p}}(F)$ can be approximated to be the force-extension curve of the long flexible linker, which follows the WLC model. With these approximations, the extrapolated zero-force relooping rates as summarized in Supplementary Table 1.

Supplementary References:

1 Chen, Z. *et al.* Programmable design of orthogonal protein heterodimers. *Nature* **565**, 106-111, doi:10.1038/s41586-018-0802-y (2019).

2 Zakeri, B. *et al.* Peptide tag forming a rapid covalent bond to a protein, through engineering a bacterial adhesin. *Proc Natl Acad Sci U S A* **109**, E690-697, doi:10.1073/pnas.1115485109 (2012).

3 Yu, M. *et al.* Effects of Mechanical Stimuli on Profilin- and Formin-Mediated Actin Polymerization. *Nano Lett* **18**, 5239-5247, doi:10.1021/acs.nanolett.8b02211 (2018).

4 Le, S. *et al.* Dystrophin As a Molecular Shock Absorber. *ACS Nano* **12**, 12140-12148, doi:10.1021/acsnano.8b05721 (2018).

5 Le, S., Yu, M. & Yan, J. Direct single-molecule quantification reveals unexpectedly high mechanical stability of vinculin-talin/alpha-catenin linkages. *Sci Adv* **5**, eaav2720, doi:10.1126/sciadv.aav2720 (2019).

6 Le, S., Yu, M. & Yan, J. Phosphorylation Reduces the Mechanical Stability of the alpha-Catenin/beta-Catenin Complex. *Angew Chem Int Ed Engl* **58**, 18663-18669, doi:10.1002/anie.201911383 (2019).

7 Le, S. *et al.* Mechanotransmission and Mechanosensing of Human alpha-Actinin 1. *Cell Rep* **21**, 2714-2723, doi:10.1016/j.celrep.2017.11.040 (2017).

8 Chen, H. *et al.* Improved high-force magnetic tweezers for stretching and refolding of proteins and short DNA. *Biophys J* **100**, 517-523, doi:10.1016/j.bpj.2010.12.3700 (2011).

9 Le, S. *et al.* Disturbance-free rapid solution exchange for magnetic tweezers single-molecule studies. *Nucleic Acids Res* **43**, e113, doi:10.1093/nar/gkv554 (2015).

10 Zhang, X. *et al.* Revealing the competition between peeled ssDNA, melting bubbles, and S-DNA during DNA overstretching by single-molecule calorimetry. *Proc Natl Acad Sci U S A* **110**, 3865-

3870, doi:10.1073/pnas.1213740110 (2013).
11 Marko, J. F. & Siggia, E. D. Stretching DNA. *Macromolecules* **28**, 8759-8770, doi:10.1021/ma00130a008 (1995).
12 Yuan, G. *et al.* Elasticity of the Transition State Leading to an Unexpected Mechanical Stabilization of Titin Immunoglobulin Domains. *Angew Chem Int Ed Engl* **56**, 5490-5493, doi:10.1002/anie.201700411 (2017).
13 Winardhi, R. S., Tang, Q., Chen, J., Yao, M. & Yan, J. Probing Small Molecule Binding to Unfolded Polyprotein Based on its Elasticity and Refolding. *Biophys J* **111**, 2349-2357, doi:10.1016/j.bpj.2016.10.031 (2016).
14 Guo, S. *et al.* Structural-elastic determination of the force-dependent transition rate of biomolecules. *Chem Sci* **9**, 5871-5882, doi:10.1039/c8sc01319e (2018).
15 Bell, G. I. Models for the specific adhesion of cells to cells. *Science* **200**, 618-627, doi:10.1126/science.347575 (1978).
